# Lipidomics Reveals Seasonal Shifts in a Large-Bodied Hibernator, the Brown Bear

**DOI:** 10.3389/fphys.2019.00389

**Published:** 2019-04-12

**Authors:** Sylvain Giroud, Isabelle Chery, Fabrice Bertile, Justine Bertrand-Michel, Georg Tascher, Guillemette Gauquelin-Koch, Jon M. Arnemo, Jon E. Swenson, Navinder J. Singh, Etienne Lefai, Alina L. Evans, Chantal Simon, Stéphane Blanc

**Affiliations:** ^1^Research Institute of Wildlife Ecology, Department of Integrative Biology and Evolution, University of Veterinary Medicine Vienna, Vienna, Austria; ^2^IPHC, University of Strasbourg, Strasbourg, France; ^3^UMR7178, CNRS, Strasbourg, France; ^4^MetaToul-LIPIDOMIQUE Core Facility, MetaboHUB, Inserm U1048, Toulouse, France; ^5^CNES, Paris, France; ^6^Department of Forestry and Wildlife Management, Inland Norway University of Applied Sciences, Koppang, Norway; ^7^Department of Wildlife, Fish and Environmental Studies, Swedish University of Agricultural Sciences, Umeå, Sweden; ^8^Faculty of Environmental Sciences and Natural Resource Management, Norwegian University of Life Sciences, Ås, Norway; ^9^Norwegian Institute for Nature Research, Trondheim, Norway; ^10^CARMEN, INSERM U1060, University of Lyon, INRA U1235, Oullins, France

**Keywords:** hibernation, body temperature, metabolism, fatty acids, glycerophospholipids, sphingomyelin, ceramide

## Abstract

Prior to winter, heterotherms retain polyunsaturated fatty acids (“PUFA”), resulting in enhanced energy savings during hibernation, through deeper and longer torpor bouts. Hibernating bears exhibit a less dramatic reduction (2–5°C) in body temperature, but lower their metabolism to a degree close to that of small hibernators. We determined the lipid composition, via lipidomics, in skeletal muscle and white adipose tissues (“WAT”), to assess lipid retention, and in blood plasma, to reflect lipid trafficking, of winter hibernating and summer active wild Scandinavian brown bears (*Ursus arctos*). We found that the proportion of monounsaturated fatty acids in muscle of bears was significantly higher during winter. During hibernation, omega-3 PUFAs were retained in WAT and short-length fatty acids were released into the plasma. The analysis of individual lipid moieties indicated significant changes of specific fatty acids, which are in line with the observed seasonal shift in the major lipid categories and can be involved in specific regulations of metabolisms. These results strongly suggest that the shift in lipid composition is well conserved among hibernators, independent of body mass and of the animals’ body temperature.

## Introduction

Lipids are found under many different forms in the organism and have pleiotropic actions in the regulation of metabolisms. In particular, dietary lipids strongly influence patterns of daily torpor and hibernation. The state of torpor corresponds to an active and drastic reduction of metabolic rate (“MR”), followed by a decrease, more or less marked, in body temperature (“T_b_”) of the animal.

Heterothermic mammals specifically select diets rich in polyunsaturated fatty acids (“PUFAs”) prior to winter. When fed diets containing plant oils that are rich in PUFAs, heterotherms exhibit a higher propensity to use torpor, they lengthen their torpor bout duration, lower their minimum T_b_, and hence increase their energy savings (Geiser and Kenagy, 1987; Frank, 1992; Florant et al., 1993; Geiser and Kenagy, 1993; Thorp et al., 1994; Bruns et al., 2000). Linoleic acid (C18:2ω6), which belongs to the omega-6 family, was often the major dietary PUFA provided. There is also evidence indicating that high amounts of dietary oleic acid (C18:1ω9) can partly (Geiser et al., 1994) or even fully (Frank and Storey, 1996) compensate for low omega-6 fatty acid intake and that this monounsaturated fatty acid (“MUFA”) also leads to increased torpor bout duration and decreased T_b_ during hibernation. However, feeding omega-6 PUFA-enriched diets did not enhance torpor in all species (Munro and Thomas, 2004) and, interestingly, diets enriched with omega-3 fatty acids appear to reduce the propensity of individuals to enter torpor and to hibernate (Hill and Florant, 2000; Giroud et al., 2018b).

Enhanced torpor expression mediated by dietary PUFAs was linked to a rise in omega-6 fatty acid content and a concomitant reduction of saturated fatty acids (“SFAs”) in lipid reserves, as well as in phospholipid (“PL”) membranes, of almost all body tissues (Ruf and Arnold, 2008). Such a remodeling of fatty acid composition in PL membranes and body tissues associated with changes in expression of torpor or hibernation was also observed independently of dietary manipulation or selection, as for instance in the deer mouse (*Peromyscus maniculatus*) (Geiser et al., 2007), in the gray mouse lemur (*Microcebus murinus*) (Giroud et al., 2009), and in free-living alpine marmots (*Marmota marmota*) (Arnold et al., 2011). In particular, for hibernators, the several months of winter hibernation correspond to long periods of fasting, relying mainly on their body fat stores. In laboratory rats, fasting resulted in a selective depletion of white adipose tissue (“WAT”) triacylglycerols in certain long-chain PUFAs, namely linolenic acid (C18:3ω3), arachidonic acid (C20:4ω6), and eicosapentaenoic acid (C20:5ω3), and relative tissue enrichment in all very long-chain SFAs and MUFAs (Raclot et al., 1995). In fasted hibernating rodents, selective fatty acids mobilization stored from triacylglycerols also occurs (Price et al., 2013). However, in contrast to non-hibernators (rats), certain unsaturated fatty acids (“USFA”), notably oleic acid (C18:1ω9) and linoleic acid (C18:2ω6), were selectively retained in the WAT of hibernating thirteen-lined ground squirrels (*Ictidomys tridecemlineatus*), while proportions of some SFAs, namely stearic acid (C18:0) and palmitic acid (16:0), were highly mobilized. MUFAs were reported to also play an important role during hibernation in some heterothermic species living in tropical and subtropical areas and that usually hibernate at higher temperatures (Falkenstein et al., 2001; Fietz et al., 2003). Echidnas and fat-tailed dwarf lemurs metabolize MUFAs during hibernation in preference over SFAs (Falkenstein et al., 2001; Fietz et al., 2003). In both species, MUFAs correspond, however, to the main proportions of total fatty acids in WAT before and after hibernation. This suggests that MUFAs can possibly compensate the low availability of essential fatty acids prior and during hibernation in tropical and sub-tropical heterothermic species. In hibernators, such changes in lipid composition are expected to ensure proper body functions at low T_b_ during torpor, possibly through the maintenance of lipid fluidity (Sinensky, 1974; Aloia and Raison, 1989; Tiku et al., 1996) and/or the regulation of membrane proteins by specific lipids (Ruf and Arnold, 2008; Giroud et al., 2013; Arnold et al., 2015).

Although the roles of fatty acids in torpor regulation have been extensively studied in small hibernators and daily heterotherms, there is, to date and to our knowledge, no systematic study on seasonal changes of lipid composition existing on large species, such as bears, which hibernate only at moderate hypothermia. Yet, owing to their low surface-to-volume ratios, bears experience particular energetic challenges, specifically lower cooling rates and an inability to rely on dropping T_b_ for MR reduction, as do small heterotherms. Also, hibernating bears do not show periodic phases of rewarming, as small hibernators typically do at regular intervals during hibernation. Therefore, their torpor bout corresponds to the entire winter hibernation period. However, bears can still reduce their metabolism to 25% of basal rates, despite regulating their T_b_ between 30° and 36°C during winter (Tøien et al., 2011). Therefore, one can expect bears to display similar, if not the same, physiological adaptations to hibernation as small hibernators. In this study, we aimed to investigate the seasonal changes in retention and mobilization of lipids from various categories, which are expected to significantly impact on metabolisms, in wild Scandinavian brown bears (*Ursus arctos*). For this purpose and because lipids are found under many different forms and have pleiotropic actions, we used a lipidomic approach to determine the lipid composition in skeletal muscle and white adipose tissues (to assess lipid retention), and in blood plasma (reflecting lipid trafficking) of bears during winter hibernation and the summer active period. Specifically, we hypothesized that bears conserve USFAs in their body tissues, and mobilize SFAs to fuel winter hibernation vs. the summer active period. Similar to tropical and subtropical heterothermic species, we expected bears to retain more specifically MUFAs to ensure their body functions during hibernation compared to when active in the summer. Further, we predicted that some specific lipid molecules are particularly mobilized or retained during winter, according to their implications in modulating the hibernation phenotype of bears.

Here we present a unique dataset assessing, for the first time, the seasonal changes of lipid composition of free-ranging brown bears (*U. arctos*) studied in their natural environment. The data are unique because the Scandinavian Brown Bear Research Project (“SBBRP”), we are part of, is the only team which has the experience of capturing free-living hibernating brown bears. The design of this study (read below for details) allowed us to determine the lipid retention/utilization in bears during the winter hibernation period, i.e., February to April (see section “Limitations of the Study” for details).

## Materials and Methods

### Study Area

The study area encompassed about 21,000 km^2^ in south-central Sweden (61°N, 15°E). The topography in this region is rolling hills, with <10% above 750 m above sea level. The area is forested and dominated by Scots pine (Pinus sylvestris L.) and Norway spruce (Picea abies H. Karst). The area is heavily used by the forestry industry, with 8% of the land clear-cut and 40% trees under 35 years of age (Moe et al., 2007). The human population is low, but there is an extensive network of forestry roads and some paved roads. The area is heavily used by hunters with dogs, not only during the moose (Alces alces) hunting season in September and October, but also during the bear hunting season, which begins on 21 August and ends when the area-specific quota has been filled, usually mid- to- late September (Swenson et al., 2017). The total population estimate for Sweden was 2,968–3,667 brown bears in 2008 (Kindberg et al., 2011). This hunting period can overlap with the pre-denning period (Evans et al., 2016). Most den abandonments occurred early in the denning season; a recent study documented that 22% of bears changed dens during winter, but only 4% after mid-December (Sahlén et al., 2015).

### Animals and Sample Collection

All personnel in the SBBRP has advanced experience and training in capturing and handling free-living brown bears during all seasons. Brown bears have been captured annually by the SBBRP and fitted with neck collars, which included a global positioning system (GPS), dual-axis motion sensors (to monitor activity), very-high-frequency (“VHF”) transmitters, and a global system for mobile mobilization (“GSM”) modem (Vectronic Aerospace GmbH, Berlin, Germany). As a backup to relocate bears if the collar malfunctioned, VHF transmitters were implanted into the abdomen (Telonics, Inc., Mesa, AZ, United States) (Arnemo et al., 2012). GPS positions were recorded every 30 min to 1 h. Bears that were the offspring of marked females were followed from birth; otherwise, age was determined by counting the annuli of a cross-section of the premolar roots (Harshyne et al., 1998). All captures and subsequent interventions carried out on the animals by trained personnel were approved by the Ethical Committee on Animal Experiments, Uppsala, Sweden (application #C47/9) and the Swedish Environmental Protection Agency. Furthermore, all experiments were performed in accordance with relevant guidelines and regulations.

Ten bears (see Table 1 for details) were used for this study. They were captured during winter hibernation in February 2011 and 2012 by darting them in their den, as previously described (Evans et al., 2012). Once anesthetized, we took each of the bears out of the winter den (during winter) and placed them on an insulated blanket. During winter, brown bears hibernate at T_b_ of ∼33°C from November to April (Evans et al., 2016). The same individuals were re-captured, when active (T_b_ ∼38°C) in June 2011 and 2012, by darting from a helicopter (Fahlman et al., 2011). The same samples were taken from these bears during both seasons. Subcutaneous WAT biopsies were obtained from only 6 individuals during the active period in summer and 5 in winter. WAT biopsies were sampled superficially to the muscle biopsies at the same surgical site. Sufficient quantities from the muscle tissue (Vastus lateralis) biopsies were available from 7 bears in summer and 8 bears in winter. Blood samples were kept in heparinized tubes at 5°C before being centrifuged within 1 h at 3,500 rpm at 5°C. Plasma and all other samples of WAT and muscle tissue were snap-frozen and stored at -80°C for subsequent lipidomic analyses.

**Table 1:** Physiological parameters of individual brown bears.

ID	Sex	Age (year)	Body Mass (kg)		Body Temperature (°C)		Tissues	
			Summer	Winter	Summer	Winter	Summer	Winter
0825	F	4	47.0	58.0	40.5	34.7	P M	P
0904	F	3	72.0	57.0	37.3	34.1	P M	P M
0908	M	3	51.0	58.0	39.9	33.4	P W M	P W M
1004	M	2	22.0	21.0	39.2	32.0	P	P M
1011	F	3	59.0	56.0	40.8	34.2	P W M	P W M
1015	M	2	27.0	25.0	38.6	33.1	P W M	P
1017	F	2	28.0	35.0	39.2	36.2	P W M	P W M
1104	F	2	29.0	30.2	39.4	32.1	P	P M
1105	F	2	–	31.5	39.4	32.0	P W	P W M
1110	F	2	29	27.3	40.0	35.1	P W M	P W M

*Animals were used in the linear mixed models (LMM) to test for the effect of season (fixed variable) on the different lipid groups or specific lipid molecules (predicted variable) in each tissue, i.e., white adipose tissue (“W”), muscle tissue (“M”), and blood plasma (“P”). Bear’s ID was included as random effect for taking repeated measurements among animals into account. Sample sizes in LMM were of 10(P), 6(W), 7(M) in summer, and of 10(P), 5(W), 8(M) in winter.*

To assess the pleiotropic actions of various lipid molecules, we performed lipidomic analyses to identify and quantify (relative quantification) five main lipid categories: total fatty acids (“FA”), sterol [i.e., free cholesterol (“C”) and esterified cholesterol (“EC”)], triacylglycerides (“TG”), glycerophospholipids (“GPL”), sphingolipids (“SL”) and cholesterol. GPL included phosphatidyl-choline (“PC”), phosphatidyl-ethanolamine (“PE”), phosphatidyl-inositol (“PI”) and phosphatidyl-serine (“PS”). SL mainly corresponded to sphingomyelin (“SM”) and ceramides (“Cer”). For each sub-category of GPL, we distinguished very long-chain (more than 20 carbons) fatty acids from medium- and long-chain fatty acids (less than 20 carbons).

### Ethics Statement

All captures and subsequent interventions carried out on the animals were approved by the Ethical Committee on Animal Experiments, Uppsala, Sweden (application #C47/9) and the Swedish Environmental Protection Agency.

### Glycerophospholipid and Ceramide-Sphingomyelin Relative Quantification

Lipids were extracted from 1 mg of WAT, 1 mg of muscle, or 10 μl of plasma by using a procedure modified from Bligh and Dyer (1959) in dichloromethane/methanol (2% acetic acid)/water (2.5:2.5:2 v/v/v) in the presence of internal standards (Cer d18:1/15:0 16 ng; PE 12:0/12:0 180 ng; PC 13:0/13:0 16 ng; SM d18:1/12:0 16 ng; PI 17:0/14:1 30 ng; PS 12:0/12:0 156.25 ng). The solution was centrifuged at 1500 rpm for 3 min. The organic phase was collected and dried under azote, then dissolved in 50 L of methanol. The extract was then stored at -20°C until subsequent analysis. Standards and sample solutions were analyzed using an Agilent 1290 Ultra Performance Liquid Chromatography (UPLC) system coupled to a G6460 triple quadrupole spectrometer (Agilent Technologies) and using “MassHunter” software for data acquisition and analysis. A Kinetex Hydrophilic Interaction Chromatography (HILIC) column (Phenomenex, 50 × 4.6 mm, 2.6 μm) was used for Liquid Chromatography (LC) separations. The column temperature was controlled at 40°C. The mobile phase A was Acetonitrile; and B was 10 mM ammonium formate in water at pH 3.2. The gradient was as follows: from 10 to 30% of B in 10 min; then 100% of B for 2 min, and then back to 10% of B at 13 min for 1-min of re-equilibration prior to the next injection. The flow rate of mobile phase was 0.3 mL/min, and the injection volume was 5μL. An electrospray source was employed in positive (for Cer, PE, PC and SM analysis) and negative ion mode (for PI and PS analysis). Azote was used as collision gas. Needle voltage was set to +4000 V. Several scan modes were used. To obtain the naturally different species’ mass, we first analyzed cells lipid extracts with a precursor ion scan of 184, 241, and 264 m/z to PC/SM, PI and Cer, respectively; and a neutral loss scan of 141 and 87 for PE and PS, respectively. The collision energy optimums for Cer, PE, PC, SM, PI, and PS were 25, 20, 30, 25, 45 and 22 eV, respectively. Then the corresponding SRM transitions were used to quantify different PL species for each class. Two class-specific positive and negative Selective Reaction Monitoring (SRM) acquisitions are necessary to account for large differences between PL classes. Data were treated using QqQ Quantitative (vB.05.00) and Qualitative analysis software (vB.04.00). For each lipid species, the relative quantification was obtained by comparing the signal derived as area under the peak for the lipid of interest with the signal resulting from its internal standard.

### Neutral Lipid Relative Quantification

We extracted lipids from 1 mg of WAT, 1 mg of muscle, 10 ml of plasma by using a procedure described by Bligh and Dyer (1959) in dichloromethane/methanol/water (2.5/2.5/2.1, v/v/v), in the presence of the internal standards : 4 μg of stigmasterol, 4 μg of cholesteryl heptadecanoate, 8 μg of glyceryl trinonadecanoate. Dichloromethane phases were evaporated to dryness and dissolved in 20 ml of ethyl acetate. 1 μl of the lipid extract was analyzed by gas-liquid chromatography on a FOCUS Thermo Electron system using Zebron-1 Phenomenex fused silica capillary columns (5 m × 0.32 mm i.d, 0.50 μm film thickness) (Barrans et al., 1994). Oven temperature was programmed from 200° to 350°C at a rate of 5°C per min and the carrier gas was hydrogen (0.5 bar). The injector and the detector temperatures were set to 315° and 345°C, respectively. For each lipid species, the relative quantification was obtained by comparing the signal derived as area under the peak for the lipid of interest with the signal resulting from its internal standard. This method allows the separation of TGs based on their total number of carbons, but does not allow structural characterization of TGs, i.e., with number and position of double bonds (Barrans et al., 1994).

### Total Fatty Acid Methyl Ester (“FAME”) Analysis

We extracted lipids from 1 mg of WAT, 1 mg of muscle, and 10 rml of plasma by using a procedure described by Bligh and Dyer (1959) in dichloromethane/methanol/water (2.5:2.5:2.1, v/v/v), in the presence of the internal standards glyceryl triheptadecanoate (2 μg). Lipid extracts were hydrolyzed in hydroxide Potassium (0.5 M in methanol) at 50°C for 30 min, and transmethylated in boron trifluoride methanol solution 14% (SIGMA, 1 ml) and heptane (1 ml) at 80°C for 1 h. After adding water (1 ml) to the crude, total FAME were extracted with heptane (3 ml), evaporated to dryness, and dissolved in ethyl acetate (20 μl). Total FAME (1 μl) were analyzed by gas-liquid chromatography (Lillington et al., 1981) on a Clarus 600 Perkin Elmer system using a Famewax RESTEK fused silica capillary columns (30 m × 0.32 mm i.d, 0.25 μm film thickness). Oven temperature was programmed from 110°to 220°C at a rate of 2°C per min and the carrier gas was hydrogen (0.5 bar). The injector and the detector temperatures were set to 225° and 245°C, respectively. For each lipid species, the relative quantification was obtained by comparing the signal derived as area under the peak for the lipid of interest with the signal resulting from its internal standard.

### Statistical Analyses

Data analyses were carried out using SAS 9.4 (SAS Institute, Inc., Cary, NC, United States). Standardized residuals from statistical models were tested for normality using Kolmogorov-Smirnov tests. We used linear mixed-effects models (“LMM”) to test for the effect of season (fixed variable) on the different lipid groups or specific lipid molecules (predicted variable), taking repeated measurements among animals into account with bear’s ID as random effect. Initial inspection of the data gave no evidence for an effect of sex or sampling year on any of predicted variables. Differences of least square means (“Lsmeans”) between seasons were assessed. To limit non-relevant results, we applied a two step-procedure: we excluded lipid species that represent very small fractions (<1%) of total lipids, because of less physiological relevance; and then corrected for multiple comparisons by considering the 5% false discovering rate (“FDR”) with the corresponding *p*-value of 0.015. Values are Lsmeans ± SE or Means ± SE and differences of Lsmeans ± SE, and *p* < 0.015 was considered significant. Analyses were performed using (1) all available samples and (2) only paired samples (10 for plasma, 5 for adipose tissue and 5 for muscle). As the results were similar, only those of the first analyses, including all available samples, are presented. Bear individuals used in the LMM are presented in Table 1.

## Results

### Lipids Levels

The level of each lipid group corresponded to the relative quantification of major lipid class, calculated as the ratio between the signal of lipids of interest and the signal of the internal standard of the lipid family to which the lipids of interest belong. Some levels of lipid groups were significantly higher in bears during winter compared to the summer active state in all three tissues (WAT, muscle, plasma). This was indeed the case for the level of total FA in WAT and plasma (Table 2 and Supplementary Table S1). However, levels of total TG in all three tissues, total FA in muscle tissue, and total PL in WAT and muscle tissue did not differ between seasons, although plasma PL levels were significantly higher during winter compared to the summer active period (Table 2 and Supplementary Table S1).

**Table 2:** Seasonal changes of concentrations of main lipid categories in brown bears.

Tissues	Variables	Means ± SE		*p*-values
		Summer	Winter	
**WAT**				
	Total FA	1.83 ± 0.84	138.89 ± 29.47	**<0.01**
	Total TG	1.00 ± 0.72	81.29 ± 43.22	0.106
	Total PL	0.10 ± 0.04	0.07 ± 0.01	0.458
**Muscle**				
	Total FA	0.10 ± 0.02	1.47 ± 1.27	0.396
	Total TG	0.01 ± 0.02	0.04 ± 0.01	0.022
	Total PL	22.87 ± 4.05	24.48 ± 2.21	0.654
**Plasma**				
	Total FA	15.10 ± 1.69	27.34 ± 1.60	**<0.01**
	Total TG	1.19 ± 0.23	6.69 ± 2.66	0.08
	Total PL	3.65 ± 0.22	5.50 ± 0.17	**<0.001**

*Arithmetic means (“Means”) and standard errors (“SE”) of concentrations (in mmol l^-*1*^) of total fatty acids (“FA”), total triacylglycerides (“TG”), and total phospholipids (“PL”) in white adipose tissue (“WAT”), muscle tissue (“Muscle”) and blood plasma (“Plasma”) of bears during the summer active period (“Summer”) and in winter hibernation (“Winter”). Sample sizes used in the linear mixed-effects models are presented in Table 1. Significant *p*-values are highlighted in bold.*

### Total Fatty Acids

During hibernation, USFAs, i.e., MUFAs and to a lower extent PUFAs, are retained in tissues, whereas SFAs seemed to be mobilized for distribution and oxidation (Supplementary Table S2). We found significantly lower MUFA-SFA, PUFA-SFA, and USFA-SFA plasma ratios in bears during winter hibernation compared to active summer (Supplementary Figure S1 and Table 3). Conversely, MUFA-SFA ratio was higher in muscle tissue in winter than in summer (Supplementary Figure S1 and Table 3). Although not significant, proportions of ω3 PUFA tended to be higher in WAT and lower in plasma in bears during winter compared to animals in summer (Table 3 and Supplementary Table S2). Specifically, plasma proportions of C18:3ω3 and C20:5ω3 were significantly reduced during winter hibernation vs. active summer (Figure 2 and Supplementary Table S2). Conversely, the proportion of C20:4ω6 in WAT was significantly increased in bears during winter compared to animals in summer (Figure 2 and Supplementary Table S2). This suggests that some specific ω3 fatty acids, namely C18:3ω3 and C20:5ω3, tend to be retained in tissue during winter hibernation, and some specific ω6 PUFA being mobilized.

**Table 3:** Seasonal changes of ratios and proportions of fatty acid groups in brown bears.

Tissues	Variables	Means ± SE		Winter – summer differences (%FA)	
		Summer	Winter	Lsmeans ± SE	*p*-values
**WAT**					
	MUFA/PUFA	6.11 ± 1.30	13.75 ± 3.47	7.86 ± 2.62	0.044
	MUFA/SFA	1.09 ± 0.13	1.55 ± 0.13	0.52 ± 0.14	0.044
	PUFA/SFA	0.20 ± 0.04	0.14 ± 0.04	-0.06 ± 0.05	0.295
	USFA/SFA	1.29 ± 0.12	1.69 ± 0.13	0.39 ± 0.20	0.182
	SFA	44.08 ± 0.02	37.49 ± 0.02	-6.51 ± 3.31	0.208
	Omega-3 [%PUFA]	18.38 ± 5.31	29.63 ± 4.42	11.76 ± 3.29	0.033
**Muscle**					
	MUFA/PUFA	4.66 ± 1.22	14.76 ± 3.76	10.75 ± 4.50	0.045
	MUFA/SFA	0.67 ± 0.10	1.39 ± 0.13	0.63 ± 0.19	**0.009**
	PUFA/SFA	0.22 ± 0.06	0.20 ± 0.08	-0.02 ± 0.10	0.815
	USFA/SFA	0.88 ± 0.06	1.59 ± 0.19	0.66 ± 0.24	0.022
	SFA	53.39 ± 0.02	39.71 ± 0.02	-13.67 ± 2.95	**<0.001**
	Omega-3 [%PUFA]	15.37 ± 2.39	11.09 ± 7.21	-3.39 ± 8.49	0.699
**Plasma**					
	MUFA/PUFA	0.97 ± 0.08	1.13 ± 0.12	0.16 ± 0.11	0.201
	MUFA/SFA	1.06 ± 0.08	0.72 ± 0.05	-0.33 ± 0.08	**0.002**
	PUFA/SFA	1.12 ± 0.07	0.69 ± 0.07	-0.44 ± 0.12	**0.006**
	USFA/SFA	2.18 ± 0.11	1.41 ± 0.09	-0.77 ± 0.18	**0.002**
	SFA	31.80 ± 0.01	41.82 ± 0.01	10.02 ± 2.34	**0.002**
	Omega-3 [%PUFA]	19.08 ± 1.85	12.90 ± 1.31	-6.18 ± 2.63	0.043

*Arithmetic means (“Means”) and standard errors (“SE”) of response variables from white adipose tissue (“WAT”), muscle tissue (“Muscle”), and blood plasma (“Plasma”) of bears during the summer active period (“Summer”) and in winter hibernation (“Winter”). Response variables are ratios between different fatty acid (“FA”) groups: between monounsaturated fatty acid (“MUFA”) and polyunsaturated fatty acid (“PUFA”), between MUFA and saturated fatty acid (“SFA”), between PUFA and SFA, between unsaturated fatty acid (“USFA”) and SFA (among total fatty acids), as well as proportions of SFA and omega-3 FA (among total PUFA). Differences of least square means (“Lsmeans”) between seasons and *p*-values result from linear-mixed effects models (LMM). Sample sizes used in the LMM are presented in Table 1. Significant *p*-values are highlighted in bold.*

### Triacylglycerides

During hibernation, TGs with the shortest fatty acids appeared to be released into the plasma, whereas those with longer chains are retained in muscle tissue. We found statistically significant 3.4- and 1.6-fold higher plasmatic proportions of TGs with chain length of either 49 carbons (“C49”) or 51 carbons (“C51”), respectively, in bears in winter than during summer (Supplementary Figure S2 and Table 4). Conversely, plasma proportions of long-chain TGs with chain length of either 55 carbons (“C55”) or 57 carbons (“C57”) were 37 and 72% lower, respectively, during winter vs. summer (Supplementary Figure S2 and Table 4). The bears showed a 1.2-fold higher proportion of TGs with chain length of 55 carbons (“C55”) in their muscle tissue during winter than when active in summer (Supplementary Figure S2 and Table 4). The WAT of bears did not show any significant seasonal changes in the proportions of different TGs.

**Table 4:** Seasonal changes of proportions of triacylglycerides in brown bears.

Tissues	Variables	Means ± SE		Winter – summer differences (%TG)	
		Summer	Winter	Lsmeans ± SE	*p*-values
**WAT**					
	C49	4.12 ± 1.19	0.43 ± 0.04	-3.69 ± 1.41	0.078
	C51	4.43 ± 0.76	5.18 ± 0.62	0.71 ± 0.75	0.405
	C53	21.17 ± 1.70	22.25 ± 0.57	1.08 ± 1.76	0.572
	C55	50.98 ± 3.09	51.85 ± 2.01	1.76 ± 2.10	0.461
	C57	18.80 ± 1.26	18.18 ± 0.82	-0.96 ± 1.21	0.482
**Muscle**					
	C49	2.80 ± 1.15	0.41 ± 0.10	-2.42 ± 1.01	0.039
	C51	5.24 ± 1.82	4.30 ± 0.46	-1.00 ± 1.66	0.563
	C53	22.62 ± 4.12	21.63 ± 0.43	-0.97 ± 3.69	0.799
	C55	42.91 ± 1.77	52.03 ± 1.17	8.70 ± 2.28	**0.006**
	C57	24.43 ± 5.63	19.44 ± 0.41	-4.98 ± 5.02	0.345
**Plasma**					
	C49	8.08 ± 2.59	27.20 ± 2.15	19.12 ± 2.82	**<0.001**
	C51	15.10 ± 1.60	23.91 ± 1.05	8.82 ± 1.46	**<0.001**
	C53	13.33 ± 2.04	19.53 ± 0.89	6.20 ± 2.57	0.039
	C55	32.15 ± 1.83	20.30 ± 1.43	-11.85 ± 1.79	**<0.001**
	C57	26.74 ± 2.17	7.65 ± 0.59	-19.09 ± 2.16	**<0.001**

*Arithmetic means (“Means”) and standard errors (“SE”) of response variables from white adipose tissue (“WAT”), muscle tissue (“Muscle”), and blood plasma (“Plasma”) of brown bears during the summer active period (“Summer”) and in winter hibernation (“Winter”). Response variables are proportions of triacylglycerides (“TG”) with different carbon chain lengths from C49 to C57. Differences of least square means (“Lsmeans”) between seasons and *p*-values result from linear-mixed effects models (LMM). Sample sizes used in the LMM are presented in Table 1. Significant *p*-values are highlighted in bold.*

### Glycerophospholipids and Sphingolipids

Seasonal changes of GPL and SL in the three tissues were minor overall. In WAT, we found no significant differences in any of GPL and SL classes from bears between summer and winter states (Supplementary Figure S3 and Table 5). Although not significant, proportions of PC and PS tended to be slightly higher (5%) and lower, respectively, in muscle tissue of bears during winter vs. summer (Supplementary Figure S3 and Table 5). In plasma, the proportion of PI showed a significant decrease of 29%, whereas the proportion of SM tended to increase by 21% in bears during winter hibernation compared to when active in summer (Supplementary Figure S3 and Table 5).

**Table 5:** Seasonal changes of different phospholipids in brown bears.

Tissues	Variables	Means ± SE		Winter – summer differences (%PL)	
		Summer	Winter	Lsmeans ± SE	*p*-values
**WAT**					
	Cer	4.90 ± 2.61	1.75 ± 1.19	-3.31 ± 3.07	0.350
	PC	68.48 ± 6.06	68.87 ± 2.06	1.48 ± 7.43	0.854
	PE	2.70 ± 0.26	6.87 ± 2.06	4.19 ± 1.75	0.074
	PI	12.19 ± 1.45	11.96 ± 4.43	-0.40 ± 4.10	0.928
	PS	1.90 ± 0.37	2.07 ± 0.33	0.12 ± 0.44	0.658
	SM	9.83 ± 2.41	8.48 ± 1.97	-1.73 ± 2.19	0.479
**Muscle**					
	Cer	0.29 ± 0.05	0.26 ± 0.02	-0.03 ± 0.06	0.618
	PC	64.06 ± 1.51	66.53 ± 1.33	3.25 ± 1.17	0.045
	PE	9.27 ± 1.03	6.67 ± 0.65	-2.64 ± 1.20	0.055
	PI	17.69 ± 1.82	17.99 ± 1.21	0.10 ± 1.86	0.958
	PS	1.26 ± 0.08	1.02 ± 0.05	-0.23 ± 0.10	0.049
	SM	7.44 ± 0.67	7.53 ± 0.56	0.12 ± 0.83	0.892
**Plasma**					
	Cer	0.30 ± 0.02	0.26 ± 0.01	-0.03 ± 0.03	0.240
	PC	69.85 ± 0.67	68.08 ± 1.45	-1.75 ± 1.74	0.339
	PE	1.04 ± 0.15	1.02 ± 0.11	-0.03 ± 0.17	0.973
	PI	8.08 ± 0.40	5.65 ± 0.32	-2.35 ± 0.34	**<0.001**
	PS	0.28 ± 0.04	0.33 ± 0.11	0.05 ± 0.13	0.702
	SM	20.46 ± 0.91	24.66 ± 1.37	4.20 ±1.72	0.036

*Arithmetic means (“Means”) and standard errors (“SE”) of response variables from white adipose tissue (“WAT”), muscle tissue (“Muscle”), and blood plasma (“Plasma”) of bears during the summer active period (“Summer”) and in winter hibernation (“Winter”). Response variables are proportion of ceramide (“Cer”), phosphatidyl-choline (“PC”), phosphatidyl-ethanolamine (“PE”), phosphatidyl-inositol (“PI”), phosphatidyl-serine (“PS”), and Sphingomyelin (“SM”) among phospholipids (“PL”). Differences of least square means (“Lsmeans”) between seasons and *p*-values result from linear-mixed effects models (LMM). Sample sizes used in the LMM are presented in Table 1. Significant *p*-values are highlighted in bold.*

**FIGURE 1:**
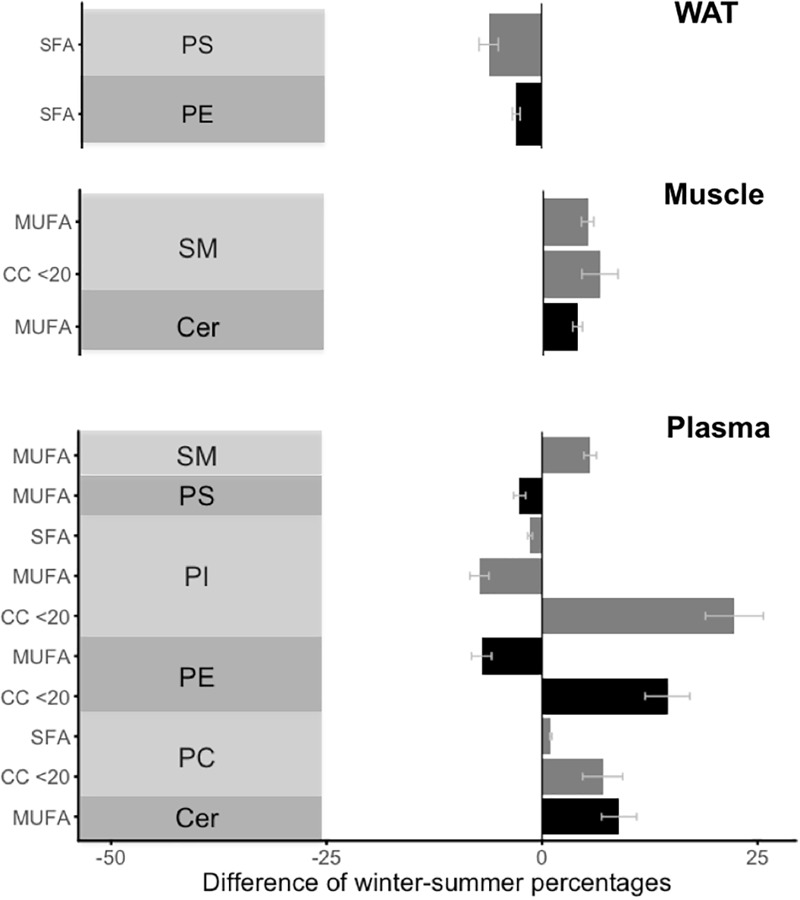
Significant (*p* < 0.015) winter-summer differences in proportion of fatty acid carbon chain length and groups of glycerophospholipids. The different glycerophospholipids include phosphatidyl-choline (“PC”), phosphatidyl-ethanolamine (“PE”), phosphatidyl-inositol (“PI”), and phosphatidyl-serine (“PS”), and sphingolipids, i.e., ceramide (“Cer”) and sphingomyelin (“SM”), in white adipose tissue (“WAT”), skeletal muscle (“Muscle”), and blood plasma (“Plasma”) from winter hibernating and summer active brown bears. For a better readability, the use of a gray or a dark bar was alternated for each lipid group. Fatty acids with less than 20 carbons (“CC < 20”) include medium- and long-chain fatty acids. Different groups are monounsaturated fatty acids (“MUFA”), polyunsaturated fatty acids (“PUFA”), and saturated fatty acids (“SFA”). Error bars represent standard errors.

Despite these minor changes, the composition of bioactive GPL showed a global trend of releasing the shortest fatty acids into the plasma. Indeed, proportions of fatty acids with less than 20 carbons (i.e., medium- and long-chain) were mainly increased in the plasma during winter hibernation compared to summer (Figure 1). Further, USFA seemed to be retained in tissues, as proportions of MUFA increased significantly in muscle-SM and muscle-Cer during winter vs. summer (Figure 1).

### Specific Lipid Moieties

**FIGURE 2:**
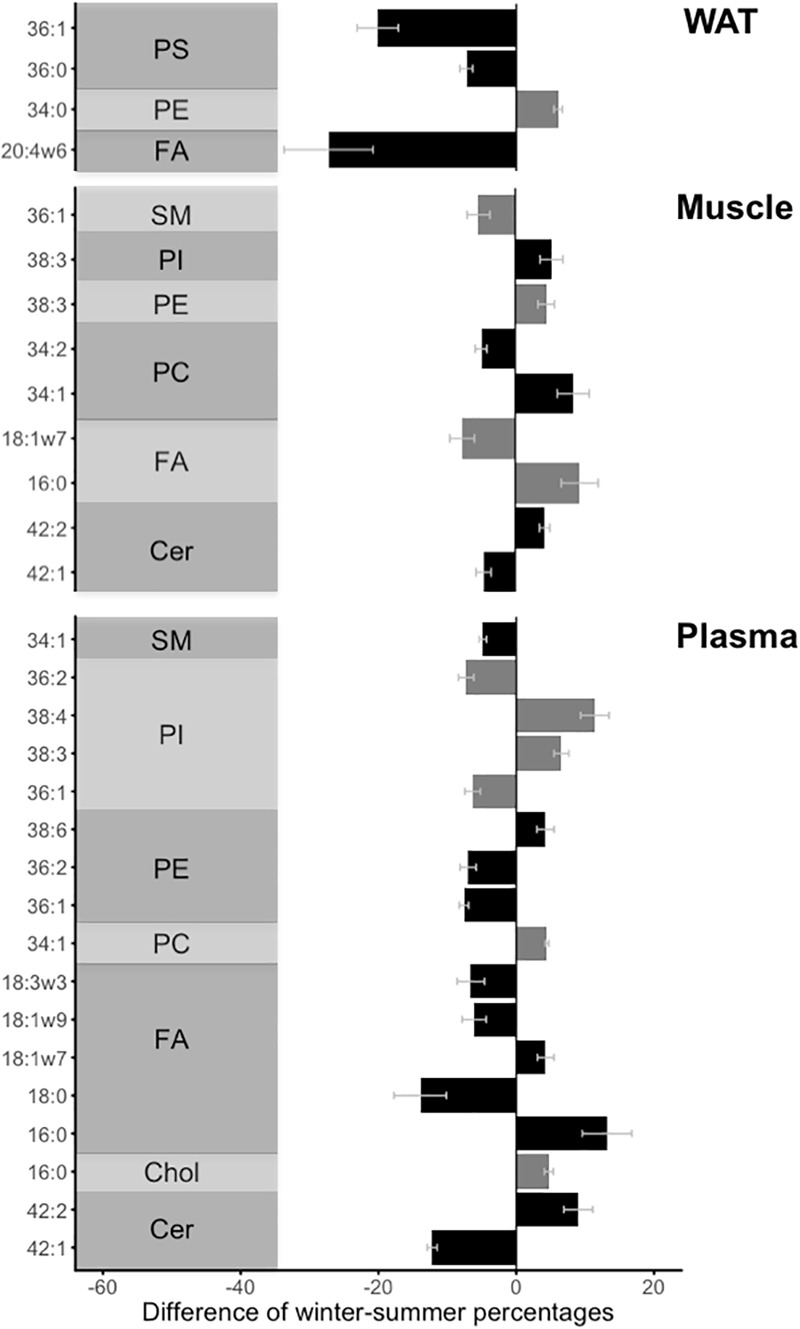
Significant (*p* < 0.015) winter-summer differences (more than 4% changes) in proportions of lipid moieties from different lipid groups. The different lipid groups include phosphatidyl-choline (“PC”), phosphatidyl-ethanolamine (“PE”), phosphatidyl-inositol (“PI”), phosphatidyl-serine (“PS”), fatty acids (“FA”) and sphingolipids, i.e., ceramide (“Cer”) and sphingomyelin (“SM”), in white adipose tissue (“WAT”), skeletal muscle (“Muscle”), and blood plasma (“Plasma”) from winter hibernating and summer active brown bears. For a better readability, the use of a gray or a dark bar was alternated for each lipid group. Error bars represent standard errors.

The different lipid moieties indicated that the proportions of specific lipid molecules varied significantly between seasons in all three tissues (Figure 2). In addition, the concentrations and relative proportions of specific fatty acids, among total FA, are presented in Supplementary Tables S1 and S2, respectively.

In Cer, proportions of the C42:1 molecule showed a significant decrease in muscle tissue and plasma during winter hibernation compared to summer. Conversely, bears showed higher proportions of C42:2 molecule in Cer both in plasma and in muscle tissue during winter. Further, the proportion of plasma cholesterol C16:0 increased in bear plasma during winter vs. summer.

Among FA, C16:0 proportion was significantly higher in bear plasma and muscle tissue during hibernation compared to summer, whereas plasma level of C18:0 was significantly lower in hibernating bears compared to animals in summer. The proportion of C18:1ω9 and C18:3ω3 in plasma were decreased in bears during winter compared to summer levels. Level of C18:1ω7 significantly increased in bear plasma during winter vs. summer, but was significantly lower in bear muscle during hibernation compared to summer. Lastly, proportion of 20:4ω6 in WAT was significantly reduced by 24% during winter, which was the largest change overall observed among FA in all three tissues.

The lipid composition of GPL and SL also showed substantial seasonal changes. In PC, plasmatic and muscular proportions of the low saturation C34:1 molecule, were significantly increased during winter, whereas the proportion of more unsaturated C34:2 molecule was lower in muscle from hibernating bears. In PE, lipid molecules of longer carbon chain, such as C38:3 in muscle and C34:0 in WAT, were retained during winter. Conversely, proportions of the high saturation C36:2 and C36:1 molecule in PE and PI were reduced in plasma during winter. In PI, proportions of highly unsaturated molecules, such as C38:4 and C38:3 in plasma and C38:3 in muscle were increased by 9.9-, 6.2-, and 5.8-fold, respectively, during winter compared to summer. In PS, proportions of low saturation molecules, such as 36:0 and 36:1, were reduced in WAT during winter. In SM, proportions of the monounsaturated molecules C36:1 in muscle and C34:1 in plasma were both lowered during winter compared to summer.

## Discussion

### Selective Retention of Unsaturated Fatty Acids in Bears During Winter

Our results showed that, during hibernation, bears specifically conserved USFAs, both MUFAs and PUFAs, in WAT and muscle tissue. These findings are in line with studies on small heterothermic mammals that tend to enrich their tissues and membrane PL with USFAs prior to hibernation. For instance, alpine marmots increase proportions of omega-6 PUFAs in their membrane PL just prior to hibernation, showing high amounts of these fatty acids in PL during winter (Arnold et al., 2011). Similarly, gray mouse lemurs (*M. murinus*) retain mainly PUFAs (i.e., C18:2ω6) in their body tissues and membranes during the winter dry season, as an increasing torpor expression in response to calorie restriction (Giroud et al., 2009). It has to be noted that the diet preferences of bears in autumn, i.e., mostly consisting of berries and low plant materials (Dahle et al., 1998; Persson et al., 2001; Stenset et al., 2016), suggest that they rely mainly, if not exclusively, on selective lipid remodeling independently of the diet to conserve USFAs during hibernation. Also, bears of our study were relatively young (2–4 years) and small in body size (Table 1). One could expect that the observed seasonal changes in lipid composition would have been partly related to postnatal development and not caused specifically by hibernation. However, in seasonal environments, young individuals, even more than adults, are subjected to strong pressures to survive their first winters in hibernation. Indeed, they have to reach an optimal fattening, both in terms of amount and quality of lipid stores, along with ensuring their development and structural growth (Arendt, 1997; Giroud et al., 2012, 2014). For instance, juveniles that were born early or late in the reproductive season, as well as sub-adult garden dormice are able, along with ensuring structural growth, to accumulate fat reserves of sufficient quantity and quality (in terms of lipid composition) already during their first winter in hibernation (Giroud et al., 2014, 2018b; Mahlert et al., 2018).

The bears from our study also tended to conserve MUFAs over PUFAs during hibernation. A similar finding was reported in deer mice, which exhibit daily torpor, mainly in the winter phenotype (Geiser et al., 2007). Indeed, deer mice under winter-like conditions had a significant 2-fold lower ratio of SFAs and MUFAs in muscle PL, compared to individuals in summer phenotype. Our results also agree with studies on hibernating species living in tropical or subtropical areas, which are usually hibernating at higher temperatures. MUFAs accounted for the vast majority (∼97%) of USFAs in WAT of free-ranging fat-tailed dwarf lemurs (*Cheirogaleus medius*) prior to hibernation (Fietz et al., 2003). Similarly, in gray mouse lemurs, MUFAs also appear to be influential for the expression of torpor at moderate (28–30°C) hypothermia, in association with the contribution of PUFAs (Vuarin et al., 2014). Hence, it seems more beneficial for heterothermic species, exhibiting only moderate hypothermia, to enrich their membranes and tissues with molecules of lower unsaturation (e.g., MUFAs) than with more unsaturated molecules (e.g., PUFAs). Further, sparing preferentially MUFAs over PUFAs in winter would be even more beneficial for bears that hibernate without rewarming periodically from hibernation over the entire winter. Indeed, periodic arousals from torpor are associated with drastic increases of MR and cause enormous production of free radicals, triggering important oxidative damages to macromolecules, cells and tissues of the organism (Carey et al., 2000; Hoelzl et al., 2016). To that respect, PUFAs and, to a less extent, MUFAs are more susceptible to oxidative stress than SFAs, and can act upon peroxidation as free radicals triggering further damages to the organism (Hulbert, 2005). Hence, heterotherms tend to balance the retention of USFAs, notably PUFAs, with the generation of oxidative stress associated with periodic arousals when increasing torpor expression [(Frank and Storey, 1995; Frank et al., 1998; Giroud et al., 2009), for review see Munro and Thomas (2004)]. Yet hibernating at high T_b_, while exposed to very low ambient temperatures, would be associated with greater energetic costs, hence oxidative stress, than hibernating at low T_b_ in a cold environment. Therefore, retaining MUFAs, over PUFAs, in membranes and tissues seems to be optimal for limiting the generation of oxidative stress while still maintaining the vital functions during torpor at moderate hypothermia, such as in hibernating bears in winter.

In the present study, we also found that, during hibernation, bears retained certain omega-3 PUFAs in WAT, while some omega-6 PUFAs appear to be mobilized. Since omega-3 PUFAs are precursors of numerous pathways delivering ATP (Weber, 2009), they have to be diverted from organs of high metabolism, such as muscles, and to be retained in non-metabolic tissues, such as WAT. This result is in line with studies on small hibernators, which drastically lower their level of omega-3 fatty acids in membrane PL of key organs, such as the heart or muscle [(Geiser et al., 2007; Arnold et al., 2011; Giroud et al., 2013), see also Arnold et al. (2015) for a review]. Notably, hibernation was incompatible with high amounts of docosahexanoic acid (C22:6ω3) in the cardiac sarcoplasmic reticulum (SR) PL of Syrian hamsters (*Mesocricetus auratus*) (Giroud et al., 2013). Further, garden dormice (*Eliomys quercinus*) fed diets rich in C22:6ω3 delayed hibernation onset and entered deep hibernation only when levels of C22:6ω3 in WAT and SR-PL had been reduced to their lowest values (Giroud et al., 2018b). Interestingly, the supplementation of diet with C22:6ω3 during winter led to a reduced use of torpor in gray mouse lemurs, which instead displayed shallow (∼33°C) hypothermia (Vuarin et al., 2016); this temperature is similar to that of hibernating bears.

### Selective Mobilization of Saturated Fatty Acids and Shortest Fatty Acids

Our results indicate that, while sparing USFAs in their tissues, bears specifically mobilized the SFAs and TGs with the shortest fatty acids, which are prone to oxidation at a lower ATP cost (for review, see Schönfeld and Wojtczak, 2016), during hibernation. Conversely, medium- and long-chain fatty acids (notably USFAs) were conserved in tissues. These results agree with findings on gray mouse lemurs that selectively mobilize palmitic acid (C16:0) for oxidation during winter, sparing C18:2ω6, along with increasing torpor use in response to food restriction (Giroud et al., 2009). Also, Geiser et al. (2007) observed that medium-and long-chain USFAs (including PUFAs) increased in the muscle of deer mice during short days, in comparison with the equinox and long days. In laboratory rats, fasting resulted in a selective depletion of adipose tissue in PUFAs and MUFAs and in a relative enrichment in all very long-chain fatty acids (Raclot et al., 1995). Also, proportions of some SFAs, i.e., stearic acid (C18:0) and palmitic acid (16:0), were highly mobilized from the WAT of hibernating thirteen-lined ground squirrels, while certain USFAs, including oleic acid (C18:1ω9) and linoleic acid (C18:2ω6), were selectively retained (Price et al., 2013). Alpine marmots selectively conserve long-chain PUFA derivatives of C18:2ω6 and C18:3ω3 in body tissues, such as heart and liver, during hibernation. Long-chain fatty acids were described to occupy the middle position ∼70% of the time, i.e., sn-2, which is less susceptible to be hydrolyzed for fatty acid mobilization, of triacylglycerol isolated from marmot WAT (Florant, 1998). It has been demonstrated that the enzyme monoacylglycerol acyltransferase (MGAT), driving the re-acetylation of sn-2-monacylglycerols, was responsible for the selective incorporation of long-chain PUFAs, such as C18:2ω6, C18:3ω3, and C22:6ω3, in the hepatic PL of neonatal rats (Xia et al., 1993). In parallel to this, the enrichment of PL with specific PUFAs and mobilizations of SFA and medium-chain fatty acids from hepatic PL were occurring (Xia et al., 1993). Such a mechanism, through the modulation of MGAT activity, could likely explain the selective retention and mobilization of specific fatty acids during hibernation, and notably the seasonal changes observed in hibernating brown bears.

In our study, more variations of lipids interestingly occurred in plasma compared to WAT and muscle tissue. This further suggests that other tissues or organs might also be involved in the retention and mobilization of lipids in hibernating bears during winter. Potential organs for retention of specific lipids include the heart, of which proper function for the maintenance of homeostasis has to be continued during hibernation, and the liver for its implication in membrane PL remodeling (as outlined above). Future investigations would need to determine the underlying molecular mechanisms of seasonal changes in lipid metabolism, including retention and mobilization among different key tissues and organs, in bears and other hibernators during winter hibernation.

### Implications and Roles of Specific Lipid Molecules

From the results of detailed lipid moieties, we found significant seasonal changes among all lipid classes. The composition of Cer showed higher proportions of 42:2 molecules at the expense of 42:1 molecules and cholesterol proportions in plasma of bears during hibernation. Plasma Cer level has been reported to be elevated in type-2 diabetic subjects and may contribute to insulin resistance through activation of inflammatory mediators, such as TNF-α (Haus et al., 2009; Chavez and Summers, 2012). Insulin sensitivity was inversely correlated with C18:0, C20:0, C24:1, and total Cer. Also, plasma TNF-α concentration was increased in type-2 diabetic subjects and correlated with increased C18:1 and C18:0 Cer subspecies (Haus et al., 2009). Recently, it has been reported that grizzly bears (*U. arctos horribilis*) showed insulin resistance during hibernation in winter, but not during the active periods in spring and fall (Rigano et al., 2017). Further, highest insulin concentrations were found to occur during hibernation in captive and wild American black bears (McCain et al., 2013). In our study, specific regulations of the Cer level and composition could have been involved in the phenomenon of insulin insensitivity of hibernating bears in winter. Cer was also shown to modify intracellular signaling pathways to slow anabolism and suppress catabolism, notably of skeletal muscles, by acting on cholesterol raft (Guenther and Edinger, 2009; Bikman and Summers, 2011; Chavez and Summers, 2012). Also, PI is known to regulate PI3-kinase activity, which is involved in numerous metabolic pathways. In particular, PI enriched with PUFA activates PKC-α, 𝜀, and δ.

Among FA, we found a significant increase in plasma and muscle proportions of C16:0, the precursor of MUFAs, as well as changes in the major MUFAs and PUFAs, involved in the functioning of PL membranes and the regulation of membrane fluidity. For instance, C18:2ω6 is a crucial omega-6 PUFA involved in the maintenance of the cardiac function during hibernation, through the maintenance of calcium homeostasis in cardiomyocytes, involving a specific mechanism of regulation of the cardiac SR calcium ATPase (Giroud et al., 2013; Arnold et al., 2015; Jastroch et al., 2016). Further, fatty acid specific trafficking between organs, such as the heart and WAT, was shown to occur in hibernating alpine marmots, concerning notably C18:2ω6, C18:3ω3, and C20:4ω6 (Arnold et al., 2011). In particular, C20:4ω6 is the preferred substrate of cyclooxygenase and therefore the most important precursor of prostaglandins (PG), which are known for their function in reproduction and thermoregulation (Ueno et al., 1982; Prendergast et al., 2002; Saito et al., 2002; Ruan et al., 2008). For instance, PGE2 infusion has been shown to cause arousal from hibernation concomitant with fever in golden-mantled ground squirrels (*Callospermophilus lateralis*) (Prendergast et al., 2002). Arnold et al. (2012) reported that PGD2 and PGE2 concentrations in the alpine marmot brain changed periodically with season and age. The availability of sufficient omega-6 PUFA, i.e., C20:4ω6, precursors for PG synthesis was apparently important in spring, when the animals become reproductively active (Arnold et al., 2012). In brown bears, levels of major eicosanoids, irrespective of their anti- and pro-inflammatory properties, are significantly reduced during winter hibernation compared to the summer active state (Giroud et al., 2018a). In particular, plasma and muscle concentrations of specific epoxyeicosatrienoic acids (EET), namely 5,6-EET and 8,9-EET, were lower in hibernating bears than in summer active individuals. EETs are known to have regulatory properties on cardiac function and cellular energy metabolism (Lee et al., 1999; Xiao et al., 2004), potentially contributing to the metabolic suppression of bears at entrance and during hibernation (Evans et al., 2016; Giroud et al., 2018a).

We also found significant seasonal changes of specific lipid moieties in GPL and SL, primary constituents of lipid membranes. Both the chain length and the number of double bonds in these acyl-chains have a major influence on the physical properties of the lipids that contain them. For instance, if C18:1ω9 is substituted for C18:0 in the sn-2 position in PC, the melting point decreases to ∼1°C and then would be liquid crystalline at or even slightly below mammalian T_b_ (Hulbert et al., 2005). As outlined above, the regulation of the cardiac SR calcium ATPase is an important mechanism for the hibernator to survive low T_b_ and metabolism during hibernation. It has been shown that the SR calcium ATPase activity was regulated, via changes in protein conformation, by the contents of both cholesterol and PE in the membrane (Yeagle, 1989). PE is essential to the correct folding of membrane protein tertiary structures (Post et al., 1995). Also, PE has been described as an important regulator and stabilizer of membranes in response to ischemia. It has been shown that incorporating N,N-dimethyl-ethanolamine in lipid membranes of neonatal rat heart myocytes resulted in a stronger attenuation of cell damage upon ischemia or metabolic inhibition (Post et al., 1995). PS can act as a co-factor to numerous signaling proteins in the cell membrane and promotes clearance of lipoproteins. Indeed, PS, as well as PE, are preferred substrates of the phospholipase A_2_ (PLA_2_) (Jaross et al., 2002). By degrading membrane PL, PLA_2_ allows the release of C20:4ω6, i.e., the reaction products of PLA_2_-mediated phospho-lipolysis. C20:4ω6 itself is the precursor of a variety of eicosanoids (as already discussed above) and is important for the promotion of phagocytosis. It was reported that an enhanced uptake of PL-modified lipoproteins by macrophages, together with a decreased serum lipoprotein in conditions with increased PLA_2_ in serum, led to an increased clearance of lipoproteins in serum and tissues (Jaross et al., 2002).

## Limitations of the Study

One possible limitation of the study can be linked to the stress and physical activity induced by capture of the bears in summer via darting them from a helicopter. Because bears in summer had to run away to try to escape, the occurrence of stress and physical activity would have possibly impacted the lipid profile (notably FFAs and possibly TGs) in plasma and, to a lesser extent, in muscle tissue. In contrast, lipids in the WAT could not have been affected by the occurrence of stress. In the SBBRP, all protocols for captures and anesthesia of bears, as performed by experienced veterinarians and field workers, are designed and optimized in order to have the less impact of stress on the physiological parameters of the animals, both in winter hibernation and during the summer active period (Evans et al., 2012; Græsli et al., 2015). In particular, pursuit and drug induction times are reduced to a minimum in order to minimize increase in T_b_, alteration of acid-base balance, and impacts on other physiological parameters in bears immobilized by remote injection, such as darting from a helicopter in summer (Cattet et al., 2003; Evans et al., 2012).

Another limitation of the study would be associated to the descriptive aspect of this work. Because of limited tissue amount that is possible to collect on bears in the field, we could not assess, in this study, more than a thorough analysis of lipid composition from specific tissues relevant for hibernation, which already constitutes a significant step. Although not mechanistic, this collaborative work constitutes, however, a unique study, because it assessed for the first time the seasonal changes of lipid composition of bears under free-living conditions. This is of major importance because laboratory diets fail to reflect natural diet selection of free-living animals that, as reported above, constrain hibernation physiology and phenology. Diet is seasonally variable in bears in Scandinavia (Persson et al., 2001; Stenset et al., 2016). In autumn, when brown bears have to build up fat reserves, berries, such as from the *Vaccinum* family, are the main food items, contributing most (49–81%) of the dietary energy content of the bears (Dahle et al., 1998; Persson et al., 2001; Stenset et al., 2016). A recent study using a ten-year time series demonstrated that greater access to bilberries improves both autumn weights of female brown bears and spring weights of yearling bears in central Sweden (Hertel et al., 2017). The intake of vegetation, a source for essential fatty acids, is of low importance in all seasons, notably prior to winter (Dahle et al., 1998; Persson et al., 2001; Stenset et al., 2016).

## Conclusion

Our study showed the interesting result that, even if the brown bear hibernates at shallow hypothermia (30–36°C), selective mobilizations and utilizations of lipids also occur, as they do in small hibernators with more pronounced T_b_ reduction during hibernation. Indeed, tissues appeared to preferentially retain MUFAs over PUFAs, and to mobilize SFAs for distribution and oxidation. Omega-3 fatty acids, precursors of numerous metabolic pathways, were sequestered in WAT. TGs with short-length fatty acids, prone to oxidation at a lower ATP cost, were released into the plasma, whereas those with longest chains were conserved in muscle tissues. The analysis of individual lipid moieties, showing the largest changes during hibernation, revealed that membrane fluidity, lipoprotein metabolism, protein conformation, i.e., 3-dimensional structure of proteins, and kinase activations were the main pathways targeted by the lipid composition of hibernating bears in winter. Clearly, further studies are needed to link lipid composition to specific functions during hibernation in bears. However, these functions might include specific regulations of, among others, the cardiovascular system (such as stabilization of heart rate), the induction and maintenance of active metabolic suppression, and the preservation of muscle mass from inactive hibernating bears in winter. Further, our results strongly suggest that, despite few differences with regard to other species, the shift in lipid composition is a conserved phenomenon of the hibernation phenotype, which seems to be independent of body mass and temperature of the animals.

## Author Contributions

SB, GG-K, JA, JS, EL, and CS initiated the study and designed the experiments. IC, FB, GT, AE, SB, and JA contributed during fieldwork and data collection. SB and JA provided the equipment. JB-M realized the lipid analyses. CS and NS performed the statistical data analysis. SG prepared the figures and drafted the manuscript. All authors participated in revisions.

## Conflict of Interest Statement

The authors declare that the research was conducted in the absence of any commercial or financial relationships that could be construed as a potential conflict of interest.

## Supplementary Material

Figure S1 Degree of unsaturation represented by ratios of different fatty acids groups (“FA ratios”). Fatty acids compositions were determined in white adipose tissue (“WAT”), skeletal muscle (“Muscle”), and blood plasma (“Plasma”) from active (“summer”) and hibernating (“winter”) brown bears. Fatty acids groups are monounsaturated fatty acids (“MUFA”), polyunsaturated fatty acids (“PUFA”), unsaturated fatty acids (“USFA”), and saturated fatty acids (“SFA”). Error bars represent standard errors. Winter levels differing significantly (*p* < 0.015) from their respective summer level are denoted by a subscript (^∗^).

Figure S2 Proportions of triacylglycerides (“TG”) – % of total TG – of different carbon chain lengths, and their respective fatty acid composition. Proportions of different TGs were determined in white adipose tissue (“WAT”), skeletal muscle (“Muscle”), and blood plasma (“Plasma”) of active (“summer”) and hibernating (“winter”) brown bears. Error bars represent standard errors. Winter levels differing significantly (*p* < 0.015) from their respective summer level are denoted by a subscript (^∗^).

Figure S3 Proportions – % of total glycerophospholipids (“GPL”) and sphingolipids (“SL”) – of different groups of GPL and SL. Proportions of ceramide (“Cer”), phosphatidyl-choline (“PC”), phosphatidyl-ethanolamine (“PE”), phosphatidyl-inositol (“PI”), phosphatidyl-serine (“PS”), and sphingomyelin (“SM”) were determined in white adipose tissue (“WAT”), skeletal muscle (“Muscle”) and blood plasma (“Plasma”) of active (“summer”) and hibernating (“winter”) brown bears. Error bars represent standard errors. Winter levels differing significantly (*p* < 0.015) from their respective summer level are denoted by a subscript (^∗^).

Table S1 Arithmetic means (“Means”) standard errors (“SE”) of concentrations (in mmol l^-1^) of specific fatty acids among total fatty acids in white adipose tissue (“WAT”), muscle tissue (“Muscle”) and blood plasma (“Plasma”) of bears during the summer active period (“Summer”) and in winter hibernation (“Winter”). Sample sizes used in the linear mixed-effects models are presented in Table 1. Significant p-values are highlighted in bold. “ND” refers to non-detectable.

Table S2 Arithmetic means (“Means”) and standard errors (“SE”) of proportions of specific fatty acids (“FA”) among saturated FA, monounsaturated FA or polyunsaturated FA in white adipose tissue (“WAT”), muscle tissue (“Muscle”) and blood plasma (“Plasma”) of bears during the summer active period (“Summer”) and in winter hibernation (“Winter”). Differences of least square means (“Lsmeans”) between seasons and p-values result from linear-mixed effects models (LMM). Sample sizes used in the LMM are presented in Table 1. Significant p-values are highlighted in bold. “ND” refers to non-detectable.

## References

[B1] Aloia R. C. Raison J. K. (1989). Membrane function in mammalian hibernation. *Biochim. Biophys. Acta* 988 123–146. 10.1016/0304-4157(89)90007-5 2642393

[B2] Arendt J. D. (1997). Adaptive intrinsic growth rates: an integration across taxa. *Q. Rev. Biol.* 72 149–177. 10.1086/419764

[B3] Arnemo J. M. Evans A. Fahlman Å. (2012). *Biomedical Protocols for Free-Ranging Brown Bears, Wolves, Wolverines and Lynx*. Norwegian: Norwegian Directorate for Nature Management.

[B4] Arnold W. Giroud S. Valencak T. G. Ruf T. (2015). Ecophysiology of omega fatty acids: a lid for every jar. *Physiology* 30 232–240. 10.1152/physiol.00047.2014 25933823

[B5] Arnold W. Kim P. Y. Allen K. G. D. Florant G. L. (2012). “Seasonal variation in brain prostaglandin D2 and E2 of marmots and *n*-6 fatty acid availability,” in *Living in a Seasonal World: Thermoregulatory and Metabolic Adaptations* eds Ruf T. Bieber C. Arnold W. Millesi E. (Heidelberg: Springer Verlag) 531–542.

[B6] Arnold W. Ruf T. Frey-Roos F. Bruns U. (2011). Diet-independent remodeling of cellular membranes precedes seasonally changing body temperature in a hibernator. *PLoS One* 6:e18641. 10.1371/journal.pone.0018641 21533242 PMC3076425

[B7] Barrans A. Collet X. Barbaras R. Jaspard B. Manent J. Vieu C. (1994). Hepatic lipase induces the formation of pre-β1 high density lipoprotein (HDL) from triacylglycerol-rich HDL2. A study comparing liver perfusion to *in vitro* incubation with lipases. *J. Biol. Chem.* 269 11572–11577. 8157689

[B8] Bikman B. T. Summers S. A. (2011). Ceramides as modulators of cellular and whole-body metabolism. *J. Clin. Invest.* 121 4222–4230. 10.1172/JCI57144 22045572 PMC3204836

[B9] Bligh E. G. Dyer W. J. (1959). A rapid method of total lipid extraction and purification. *Can. J. Biochem. Phys.* 37 911–917. 10.1139/o59-099 13671378

[B10] Bruns U. Frey-Roos F. Pudritz S. Tataruch F. Ruf T. Arnold W. (2000). “Essential fatty acids: their impact on free-living alpine marmots (*Marmota marmota*),” in *Life in the Cold IV* eds Heldmaier G. Klingenspor M. (New York, NY: Springer) 215–222.

[B11] Carey H. V. Frank C. L. Seifert J. P. (2000). Hibernation induces oxidative stress and activation of NF-κB in ground squirrel intestine. *J. Comp. Physiol. B* 170 551–559. 10.1007/s003600000135 11128446

[B12] Cattet M. R. L. Christison K. Caulkett N. A. Stenhouse G. B. (2003). Physiologic responses of grizzly bears to different methods of capture. *J. Wildl. Dis.* 39 649–654. 10.7589/0090-3558-39.3.649 14567227

[B13] Chavez J. A. Summers S. A. (2012). A ceramide-centric view of insulin resistance. *Cell Metab.* 15 585–594. 10.1016/j.cmet.2012.04.002 22560211

[B14] Dahle B. Sorensen O. J. Wedul E. H. Swenson J. E. Sandegren F. (1998). The diet of brown bears *Ursus arctos* in central Scandinavia: effect of access to free-ranging domestic sheep *Ovis aries*. *Wildl. Biol.* 4 147–158. 10.2981/wlb.1998.017

[B15] Evans A. L. Sahlén V. Støen O. G. Fahlman A. Brunberg S. Madslien K. (2012). Capture, anesthesia, and disturbance of free-ranging brown bears (*Ursus arctos*) during hibernation. *PLoS One* 7:e40520. 10.1371/journal.pone.0040520 22815757 PMC3398017

[B16] Evans A. L. Singh N. J. Friebe A. Arnemo J. M. Laske T. G. Fröbert O. (2016). Drivers of hibernation in the brown bear. *Front. Zool.* 13:7. 10.1186/s12983-016-0140-6 26870151 PMC4750243

[B17] Fahlman Å. Arnemo J. M. Swenson J. E. Pringle J. Brunberg S. Nyman G. (2011). Physiologic evaluation of capture and anesthesia with medetomidine–zolazepam–tiletamine in brown bears (*Ursus arctos*). *J. Zoo Wildl. Med.* 42 1–11. 10.1638/2008-0117.1 22946363

[B18] Falkenstein F. Körtner G. Watson K. Geiser F. (2001). Dietary fats and body lipid composition in relation to hibernation in free-ranging echidnas. *J. Comp. Physiol. B* 171 189–194. 10.1007/s003600000157 11352101

[B19] Fietz J. Tataruch F. Dausmann K. H. Ganzhorn J. U. (2003). White adipose tissue composition in the free-ranging fat-tailed dwarf lemur (*Cheirogaleus medius*; Primates), a tropical hibernator. *J. Comp. Physiol. B* 173 1–10. 10.1007/s00360-002-0300-1 12592437

[B20] Florant G. L. (1998). Lipid metabolism in hibernators: the importance of essential fatty acids. *Am. Zool.* 38 331–340. 10.1093/icb/38.2.331

[B21] Florant G. L. Hester L. Ameenuddin S. Rintoul D. A. (1993). The effect of a low essential fatty acid diet on hibernation in marmots. *Am. J. Physiol.* 264 R747–R753. 10.1152/ajpregu.1993.264.4.R747 8476117

[B22] Frank C. L. (1992). The influence of dietary fatty acids on hibernation by golden-mantled ground squirrels (*Spermophilus lateralis*). *Physiol. Zool.* 65 906–920. 10.1086/physzool.65.5.30158549

[B23] Frank C. L. Dierenfeld E. S. Storey K. B. (1998). The relationship between lipid peroxidation, hibernation, and food selection in mammals. *Am. Zool.* 38 341–349. 10.1093/icb/38.2.341

[B24] Frank C. L. Storey K. B. (1995). The optimal depot fat composition for hibernation by golden-mantled ground squirrels (*Spermophilus lateralis*). *J. Comp. Physiol. B* 164 536–542. 10.1007/BF00261394 7884064

[B25] Frank C. L. Storey K. B. (1996). “The effect of total unsaturate content on hibernation,” in *Adaptations to the Cold. Tenth International Hibernation Symposium* eds Geiser F. Hulbert A. J. Nicol S. C. (Armidale: University Press of New England) 211–216.

[B26] Geiser F. Kenagy G. J. (1987). Polyunsaturated lipid diet lengthens torpor and reduces body temperature in a hibernator. *Am. J. Physiol. Reg. Int. Comp. Physiol.* 252 R897–R901. 10.1152/ajpregu.1987.252.5.R897 3578556

[B27] Geiser F. Kenagy G. J. (1993). Dietary fats and torpor patterns in hibernating ground squirrels. *Can. J. Zool.* 71 1182–1185. 10.1139/z93-161 18513150

[B28] Geiser F. McAllan B. M. Kenagy G. J. (1994). The degree of dietary fatty acid unsaturation affects torpor patterns and lipid composition of a hibernator. *J. Comp. Physiol. B* 164 299–305. 10.1007/BF00346446 7962785

[B29] Geiser F. McAllan B. M. Kenagy G. J. Hiebert S. M. (2007). Photoperiod affects daily torpor and tissue fatty acid composition in deer mice. *Naturwissenschaften* 94 319–325. 10.1007/s00114-006-0193-z 17160415

[B30] Giroud S. Evans A. L. Chery I. Bertile F. Tascher G. Bertrand-Michel J. (2018a). Seasonal changes in eicosanoid metabolism in the brown bear. *Sci. Nat.* 105:58. 10.1007/s00114-018-1583-8 30291454 PMC6182652

[B31] Giroud S. Stalder G. Gerritsmann H. Kübber-Heiss A. Kwak J. Arnold W. (2018b). Dietary lipids affect the onset of hibernation in the garden dormouse (*Eliomys quercinus*): implications for cardiac function. *Front. Physiol.* 9:1235. 10.3389/fphys.2018.01235 30279661 PMC6153335

[B32] Giroud S. Frare C. Strijkstra A. Boerema A. Arnold W. Ruf T. (2013). Membrane phospholipid fatty acid composition regulates cardiac SERCA activity in a hibernator, the Syrian hamster (*Mesocricetus auratus*). *PLoS One* 8:e63111. 10.1371/journal.pone.0063111 23650545 PMC3641109

[B33] Giroud S. Perret M. Gilbert C. Zahariev A. Goudable J. Le Maho Y. (2009). Dietary palmitate and linoleate oxidations, oxidative stress, and DNA damage differ according to season in mouse lemurs exposed to a chronic food deprivation. *Am. J. Physiol. Reg. Int. Comp. Physiol.* 297 R950–R959. 10.1152/ajpregu.00214.2009 19625694

[B34] Giroud S. Turbill C. Ruf T. (2012). “Torpor use and body mass gain during pre-hibernation in late-born juvenile garden dormice exposed to food shortage,” in *Living in a Seasonal World. Thermoregulatory and Metabolic Adaptations* eds Ruf T. Bieber C. Arnold W. Millesi E. (Berlin: Springer) 481–491. 10.1007/978-3-642-28678-0_42

[B35] Giroud S. Zahn S. Criscuolo F. Chery I. Blanc S. Turbill C. (2014). Late-born intermittently fasted juvenile garden dormice use torpor to grow and fatten prior to hibernation: consequences for ageing processes. *Proc. R. Soc. B Biol. Sci.* 281:20141131. 10.1098/rspb.2014.1131 25377448 PMC4240977

[B36] Græsli A. R. Evans A. L. Fahlman Å. Bertelsen M. F. Blanc S. Arnemo J. M. (2015). Seasonal variation in haematological and biochemical variables in free-ranging subadult brown bears (*Ursus arctos*) in Sweden. *BMC Vet. Res.* 11:301. 10.1186/s12917-015-0615-2 26646442 PMC4673763

[B37] Guenther G. G. Edinger A. L. (2009). A new take on ceramide. Starving cells by cutting off the nutrient supply. *Cell Cycle* 8 1122–1126. 10.4161/cc.8.8.8161 19282666

[B38] Harshyne W. A. Diefenbach D. R. Alt G. L. Matson G. M. (1998). Analysis of error from cementum-annuli age estimates of known-age Pennsylvania black bears. *J. Wildl. Manage.* 62 1281–1291. 10.2307/3801992

[B39] Haus J. M. Kashyap S. R. Kasumov T. Zhang R. L. Kelly K. R. DeFronzo R. A. (2009). Plasma ceramides are elevated in obese subjects with type 2 diabetes and correlate with the severity of insulin resistance. *Diabetes* 58 337–343. 10.2337/db08-1228 19008343 PMC2628606

[B40] Hertel A. G. Bischof R. Langval O. Mysterud A. Kindberg J. Swenson J. E. (2017). Berry production drives bottom–up effects on body mass and reproductive success in an omnivore. *Oikos* 127 197–207. 10.1111/oik.04515

[B41] Hill V. L. Florant G. L. (2000). The effect of a linseed oil diet on hibernation in yellow-bellied marmots (*Marmota flaviventris*). *Physiol. Behav.* 68 431–437. 10.1016/s0031-9384(99)00177-8 10713281

[B42] Hoelzl F. Cornils J. S. Smith S. Moodley Y. Ruf T. (2016). Telomere dynamics in free-living edible dormice (*Glis glis*): the impact of hibernation and food supply. *J. Exp. Biol.* 219(Pt 16) 2469–2474. 10.1242/jeb.140871 27535986 PMC5004978

[B43] Hulbert A. J. (2005). On the importance of fatty acid composition of membranes for aging. *J. Theor. Biol.* 234 277–288. 10.1016/j.jtbi.2004.11.024 15757684

[B44] Hulbert A. J. Turner N. Storlien L. H. Else P. L. (2005). Dietary fats and membrane function: implications for metabolism and disease. *Biol. Rev.* 80 155–169. 10.1007/s00360-005-0025-z 15727042

[B45] Jaross W. Eckey R. Menschikowski M. (2002). Biological effects of secretory phospholipase A2 group IIA on lipoproteins and in atherogenesis. *Eur. J. Clin. Invest.* 32 383–393. 10.1046/j.1365-2362.2002.01000.x 12059982

[B46] Jastroch M. Giroud S. Barrett P. Geiser F. Heldmaier G. Herwig A. (2016). Seasonal Control of Mammalian Energy Balance: recent advances in the understanding of daily torpor and hibernation. *J. Neuroendocrinol.* 28. 10.1111/jne.12437 27755687

[B47] Kindberg J. Swenson J. E. Ericsson G. Bellemain E. Miquel C. Taberlet P. (2011). Estimating population size and trends of the Swedish brown bear *Ursus arctos* population. *Wildl. Biol.* 17 114–123. 10.2981/10-100

[B48] Lee H.-C. Lu T. Weintraub N. L. VanRollins M. Spector A. A. Shibata E. F. (1999). Effects of epoxyeicosatrienoic acids on the cardiac sodium channels in isolated rat ventricular myocytes. *J. Physiol.* 519 153–168. 10.1111/j.1469-7793.1999.0153o.x 10432346 PMC2269481

[B49] Lillington J. M. Trafford D. J. H. Makin H. L. J. (1981). A rapid and simple method for the esterification of fatty acids and steroid carboxylic acids prior to gas-liquid chromatography. *Clin. Chim. Acta* 111 91–98. 10.1016/0009-8981(81)90425-3 7226543

[B50] Mahlert B. Gerritsmann H. Stalder G. Ruf T. Zahariev A. Blanc S. (2018). Implications of being born late in the active season for growth, fattening, torpor use, winter survival and fecundity. *eLife* 7:e31225. 10.7554/eLife.31225 29458712 PMC5819945

[B51] McCain S. Ramsay E. Kirk C. (2013). The effects of hibernation and captivity on glucose metabolism and thyroid hormones in American Black Bear (*Ursus americanus*). *J. Zoo Wildl. Med.* 44 324–332. 10.1638/2012-0146R1.1 23805551

[B52] Moe T. F. Kindberg J. Jansson I. Swenson J. E. (2007). Importance of diel behaviour when studying habitat selection: examples from female Scandinavian brown bears (*Ursus arctos*). *Can. J. Zool.* 85 518–525. 10.1139/Z07-034

[B53] Munro D. Thomas D. W. (2004). The role of polyunsaturated fatty acids in the expression of torpor by mammals: a review. *Zoology* 107 29–48. 10.1016/j.zool.2003.12.001 16351926

[B54] Persson I. L. Wikan S. Swenson J. E. Mysterud A. (2001). The diet of the brown bear *Ursus arctos* in the Pasvik Valley, northeastern Norway. *Wildl. Biol.* 7 27–37. 10.2981/wlb.2001.006

[B55] Post J. A. Verkleij A. J. Langer G. A. (1995). Organization and function of sarcolemmal phospholipids in control and ischemic-reperfused cardiomyocytes. *J. Mol. Cell. Cardiol.* 27 749–760. 10.1016/0022-2828(95)90080-2 7776380

[B56] Prendergast B. J. Freeman D. A. Zucker I. Nelson R. J. (2002). Periodic arousal from hibernation is necessary for initiation of immune responses in ground squirrels. *Am. J. Physiol. Reg. Int. Comp. Physiol.* 282 R1054–R1082. 10.1152/ajpregu.00562.2001 11893609

[B57] Price E. R. Armstrong C. Guglielmo C. G. Staples J. F. (2013). Selective mobilization of saturated fatty acids in isolated adipocytes of hibernating 13-lined ground squirrels *Ictidomys tridecemlineatus*. *Physiol. Biochem. Zool.* 86 205–212. 10.1086/668892 23434780

[B58] Raclot T. Mioskowski E. Bach A. C. Groscolas R. (1995). Selectivity of fatty acid mobilization: a general metabolic feature of adipose tissue. *Am. J. Physiol.* 269 R1060–R1067. 10.1152/ajpregu.1995.269.5.R1060 7503292

[B59] Rigano K. S. Gehring J. L. Evans Hutzenbiler B. D. Chen A. V. Nelson O. L. Vella C. A. (2017). Life in the fat lane: seasonal regulation of insulin sensitivity, food intake, and adipose biology in brown bears. *J. Comp. Physiol. B* 187 649–676. 10.1007/s00360-016-1050-9 27987017

[B60] Ruan Y. C. Wang Z. Du J. Y. Zuo W. L. Guo J. H. Zhang J. (2008). Regulation of smooth muscle contractility by the epithelium in rat vas deferens: role of ATP-induced release of PGE2. *J. Physiol.* 586 4843–4857. 10.1113/jphysiol.2008.154096 18755753 PMC2614070

[B61] Ruf T. Arnold W. (2008). Effects of polyunsaturated fatty acids on hibernation and torpor: a review and hypothesis. *Am. J. Physiol. Reg. Int. Comp. Physiol.* 294 R1044–R1052. 10.1152/ajpregu.00688.2007 18171691

[B62] Sahlén V. Friebe A. Sæbø S. Swenson J. E. Støen O.-G. (2015). Den entry behavior in Scandinavian brown bears: implications for preventing human injuries. *J. Wildl. Manage.* 79 274–287. 10.1002/jwmg.822 25866420 PMC4383655

[B63] Saito S. Tsuda H. Michimata T. (2002). Prostaglandin D2 and reproduction. *Am. J. Reprod. Immunol.* 47 295–302. 10.1034/j.1600-0897.2002.01113.x 12148545

[B64] Schönfeld P. Wojtczak L. (2016). Short- and medium-chain fatty acids in energy metabolism: the cellular perspective. *J. Lipid Res.* 57 943–954. 10.1194/jlr.R067629 27080715 PMC4878196

[B65] Sinensky M. (1974). Homeoviscous adaptation - a homeostatic process that regulates the viscosity of membrane lipids in *Escherichia coli*. *Proc. Natl. Acad. Sci. U.S.A.* 71 522–525. 10.1073/pnas.71.2.522 4360948 PMC388039

[B66] Stenset N. E. Lutnæs P. N. Bjarnadóttir V. Dahle B. Fossum K. H. Jigsved P. (2016). Seasonal and annual variation in the diet of brown bears *Ursus arctos* in the boreal forest of Southcentral Sweden. *Wildl. Biol.* 22 107–116. 10.2981/wlb.00194

[B67] Swenson J. E. Schneider M. Zedrosser A. Soderberg A. Franzen R. Kindberg J. (2017). Challenges of managing a European brown bear population; lessons from Sweden, 1943-2013. *Wildl. Biol.* 1:wlb.00251 10.2981/wlb.00251

[B68] Thorp C. R. Ram P. K. Florant G. L. (1994). Diet alters metabolic rate in the yellow-bellied marmot (*Marmota flaviventris*) during hibernation. *Physiol. Zool.* 67 1213–1229. 10.2307/30163890

[B69] Tiku P. E. Gracey A. Y. Macartney A. I. Beynon R. J. Cossins A. R. (1996). Cold-induced expression of Δ9-desaturase in carp by transcriptional and posttranslational mechanisms. *Science* 271 815–818. 10.1126/science.271.5250.815 8629000

[B70] Tøien Ø. Blake J. Edgar D. M. Grahn D. A. Heller H. C. Barnes B. M. (2011). Hibernation in black bears: independence of metabolic suppression from body temperature. *Science* 331 906–909. 10.1126/science.1199435 21330544

[B71] Ueno R. Narumiya S. Ogorochi T. Nakayama T. Ishikawa Y. Hayaishi O. (1982). Role of prostaglandin D2 in the hypothermia of rats caused by bacterial lipopolysaccharide. *Proc. Natl. Acad. Sci. U.S.A.* 79 6093–6097. 10.1073/pnas.79.19.6093 6964402 PMC347059

[B72] Vuarin P. Henry P.-Y. Guesnet P. Alessandri J.-M. Aujard F. Perret M. (2014). Shallow hypothermia depends on the level of fatty acid unsaturation in adipose and liver tissues in a tropical heterothermic primate. *J. Therm. Biol.* 43 81–88. 10.1016/j.jtherbio.2014.05.002 24956961

[B73] Vuarin P. Henry P. Y. Perret M. Pifferi F. (2016). Dietary supplementation with n-3 polyunsaturated fatty acids reduces torpor use in a tropical daily heterotherm. *Physiol. Biochem. Zool.* 89 536–545. 10.1086/688659 27792535

[B74] Weber J. M. (2009). The physiology of long-distance migration: extending the limits of endurance metabolism. *J. Exp. Biol.* 212 593–597. 10.1242/jeb.015024 19218508

[B75] Xia T. Mostafa N. Bhat B. G. Florant G. L. Coleman R. A. (1993). Selective retention of essential fatty acids: the role of hepatic monoacylglycerol acyltransferase. *Am. J. Physiol.* 265 R414–R419. 10.1152/ajpregu.1993.265.2.R414 8368397

[B76] Xiao Y.-F. Huang L. Morgan J. P. (2004). Cytochrome P450: a novel system modulating Ca2+ channels and contraction in mammalian heart cells. *J. Physiol.* 508 777–792. 10.1111/j.1469-7793.1998.777bp.x 9518732 PMC2230927

[B77] Yeagle P. L. (1989). Lipid regulation of cell membrane structure and function. *FASEB J.* 3 1833–1842. 10.1096/fasebj.3.7.2469614 2469614

