# Supplementary figures and images for: Lipidomics Reveals Seasonal Shifts in a Large-Bodied Hibernator, the Brown Bear
Source: Front Physiol. 2019 Apr 12;10:389. doi: 10.3389/fphys.2019.00389 (PMC6474398; doi:10.3389/fphys.2019.00389)

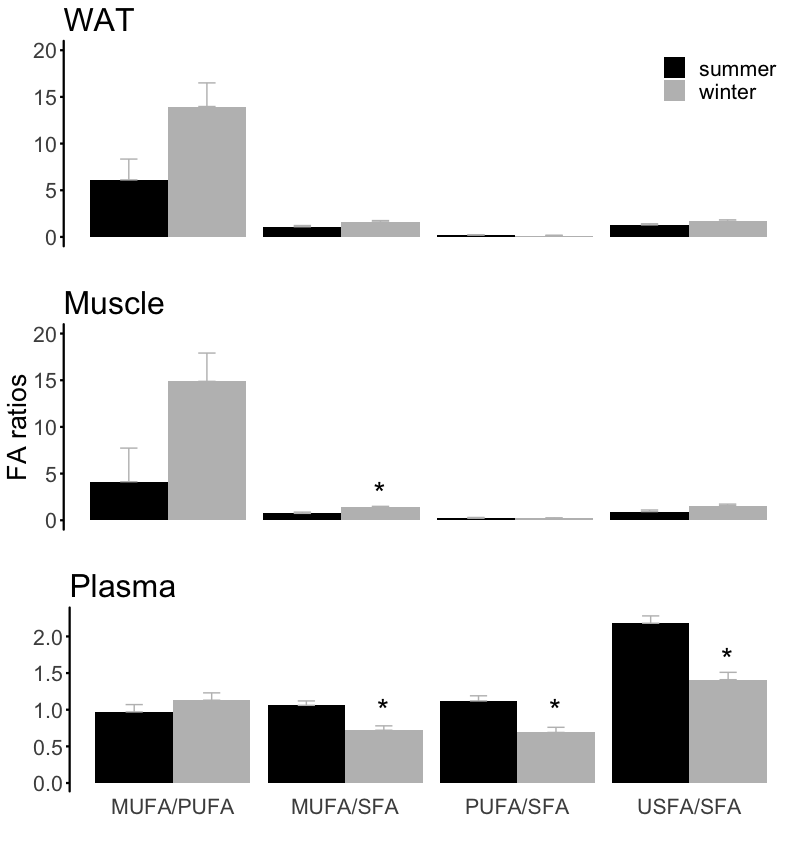

Supplement: Figure S1 — Degree of unsaturation represented by ratios of different fatty acids groups (“FA ratios”). Fatty acids compositions were determined in white adipose tissue (“WAT”), skeletal muscle (“Muscle”), and blood plasma (“Plasma”) from active (“summer”) and hibernating (“winter”) brown bears. Fatty acids groups are monounsaturated fatty acids (“MUFA”), polyunsaturated fatty acids (“PUFA”), unsaturated fatty acids (“USFA”), and saturated fatty acids (“SFA”). Error bars represent standard errors. Winter levels differing significantly (p < 0.015) from their respective summer level are denoted by a subscript (∗). [file Image_1.TIFF]

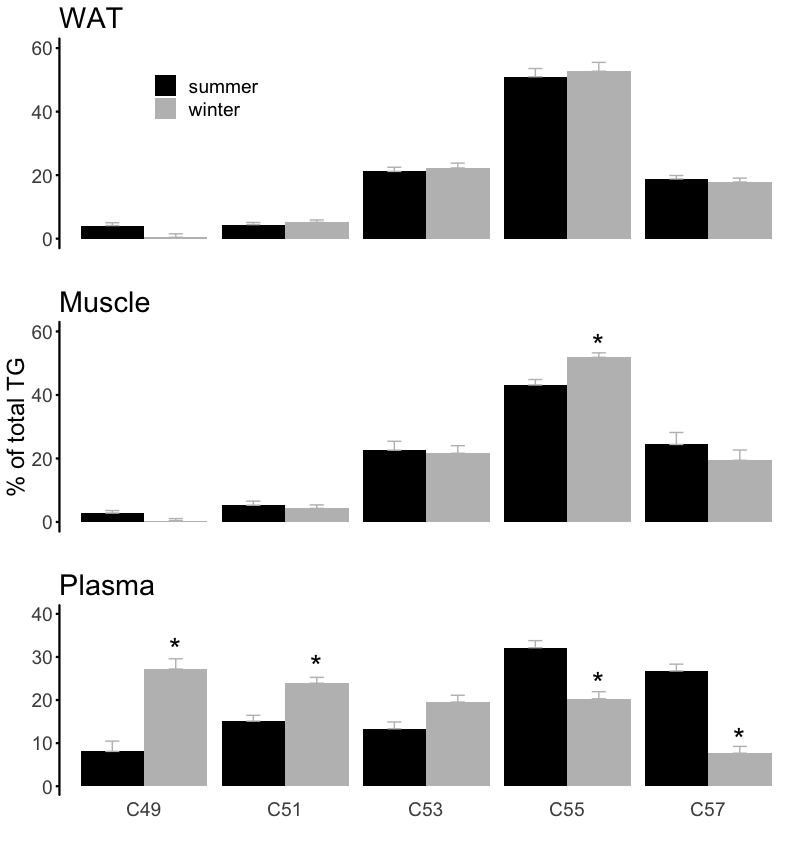

Supplement: Figure S2 — Proportions of triacylglycerides (“TG”) – % of total TG – of different carbon chain lengths, and their respective fatty acid composition. Proportions of different TGs were determined in white adipose tissue (“WAT”), skeletal muscle (“Muscle”), and blood plasma (“Plasma”) of active (“summer”) and hibernating (“winter”) brown bears. Error bars represent standard errors. Winter levels differing significantly (p < 0.015) from their respective summer level are denoted by a subscript (∗). [file Image_2.TIFF]

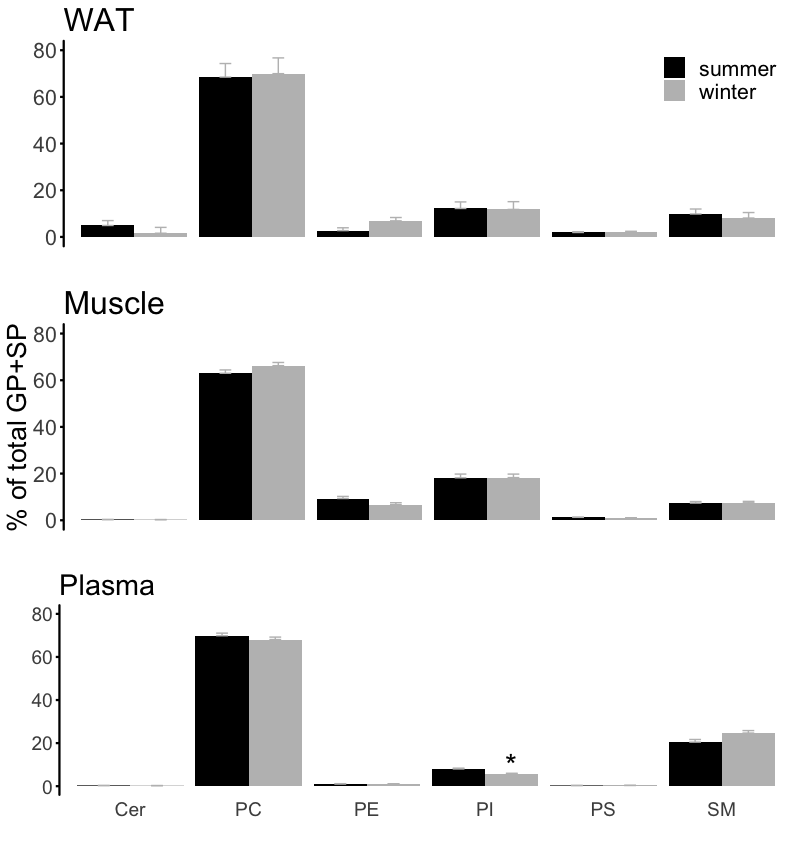

Supplement: Figure S3 — Proportions – % of total glycerophospholipids (“GPL”) and sphingolipids (“SL”) – of different groups of GPL and SL. Proportions of ceramide (“Cer”), phosphatidyl-choline (“PC”), phosphatidyl-ethanolamine (“PE”), phosphatidyl-inositol (“PI”), phosphatidyl-serine (“PS”), and sphingomyelin (“SM”) were determined in white adipose tissue (“WAT”), skeletal muscle (“Muscle”) and blood plasma (“Plasma”) of active (“summer”) and hibernating (“winter”) brown bears. Error bars represent standard errors. Winter levels differing significantly (p < 0.015) from their respective summer level are denoted by a subscript (∗). [file Image_3.TIFF]
