# Supplementary material for: Lipidomics Reveals Seasonal Shifts in a Large-Bodied Hibernator, the Brown Bear
Source: Front Physiol. 2019 Apr 12;10:389. doi: 10.3389/fphys.2019.00389 (PMC6474398; doi:10.3389/fphys.2019.00389)
Supplement: Table S1 — Arithmetic means (“Means”) standard errors (“SE”) of concentrations (in mmol l-1) of specific fatty acids among total fatty acids in white adipose tissue (“WAT”), muscle tissue (“Muscle”) and blood plasma (“Plasma”) of bears during the summer active period (“Summer”) and in winter hibernation (“Winter”). Sample sizes used in the linear mixed-effects models are presented in Table 1. Significant p-values are highlighted in bold. “ND” refers to non-detectable. [file Table_1.DOCX]

**SUPPLEMENTARY INFORMATION**

TITLE:

LIPIDOMICS REVEALS SEASONAL SHIFTS IN A LARGE-BODIED HIBERNATOR, THE BROWN BEAR

AUTHORS:

Sylvain Giroud, Isabelle Chery, Fabrice Bertile, Justine Bertrand-Michel, Georg Tascher, Guillemette Gauquelin-Koch, Jon M Arnemo, Jon E. Swenson, Navinder J. Singh, Etienne Lefai, Alina L. Evans, Chantal Simon, Stéphane Blanc

Table S1: Arithmetic means (‘Means’) standard errors (‘SE’) of concentrations (in mmol l^-1^) of specific fatty acids among total fatty acids in white adipose tissue (‘WAT’), muscle tissue (‘Muscle’) and blood plasma (‘Plasma’) of bears during the summer active period (‘Summer’) and in winter hibernation (‘Winter’). Sample sizes used in the linear mixed-effects models are presented in Table 1. Significant p-values are highlighted in bold. ‘ND’ refers to non-detectable.

| Tissues | Fatty acids | Means ± SE | |  | P-values |
| --- | --- | --- | --- | --- | --- |
|  |  | **Summer** | **Winter** |  |  |
| WAT |  |  |  |  |  |
|  | C10:0 | ND | ND |  |  |
|  | C12:0 | ND | ND |  |  |
|  | C14:0 | 0.01 ± 0.01 | 1.39 ± 0.26 |  | **<0.01** |
|  | C15:0 | ND | ND |  |  |
|  | C16:0 | 0.47 ± 0.20 | 37.65 ± 9.05 |  | **0.01** |
|  | C18:0 | 0.26 ± 0.09 | 11.89 ± 2.34 |  | **<0.01** |
|  | C20:0 | 0.01 ± 0.01 | 0.29 ± 0.02 |  | **<0.001** |
|  | C21:0 | ND | ND |  |  |
|  | C22:0 | 0.00 ± 0.01 | 0.07 ± 0.07 |  | 0.328 |
|  | C23:0 | ND | ND |  |  |
|  | C24:0 | ND | ND |  |  |
|  | C14:1 ω5 | ND | ND |  |  |
|  | C15:1 ω5 | ND | ND |  |  |
|  | C16:1 ω7 | 0.01 ± 0.01 | 0.37 ± 0.09 |  | **0.011** |
|  | C16:1 ω9 | 0.06 ± 0.03 | 8.50 ± 2.08 |  | **0.01** |
|  | C17:1 ω7 | ND | ND |  |  |
|  | C18:1 ω7 | 0.06 ± 0.03 | 3.57 ± 0.93 |  | **0.013** |
|  | C18:1 ω9 | 0.81 ± 0.42 | 67.14 ± 10.95 |  | **<0.01** |
|  | C20:1 ω9 | 0.01 ± 0.01 | 1.03 ± 0.24 |  | **<0.01** |
|  | C22:1 ω9 | ND | ND |  |  |
|  | C24:1 ω9 | ND | ND |  |  |
|  | C18:2 ω6 | 0.07 ± 0.03 | 4.13 ± 1.23 |  | 0.02 |
|  | C20:2 ω6 | 0.00 ± 0.00 | 0.26 ± 0.12 |  | 0.071 |
|  | C22:2 ω6 | ND | ND |  |  |
|  | C18:3 ω3 | 0.02 ± 0.01 | 1.42 ± 0.56 |  | 0.048 |
|  | C18:3 ω6 | ND | ND |  |  |
|  | C20:3 ω3 | 0.00 ± 0.00 | 0.03 ± 0.03 |  | 0.328 |
|  | C20:3 ω6 | 0.00 ± 0.00 | 0.09 ± 0.02 |  | **<0.01** |
|  | C20:4 ω6 | 0.04 ± 0.01 | 0.20 ± 0.02 |  | **0.011** |
|  | C22:4 ω6 | 0.01 ± 0.01 | 0.13 ± 0.01 |  | 0.049 |
|  | C20:5 ω3 | 0.01 ± 0.01 | 0.12 ± 0.05 |  | 0.071 |
|  | C22:5 ω3 | 0.01 ± 0.01 | 0.35 ± 0.12 |  | 0.027 |
|  | C22:5 ω6 | ND | ND |  |  |
|  | C22:6 ω3 | 0.01 ± 0.01 | 0.25 ± 0.24 |  | 0.308 |
| Muscle |  |  |  |  |  |
|  | C10:0 | ND | ND |  |  |
|  | C12:0 | ND | ND |  |  |
|  | C14:0 | ND | ND |  |  |
|  | C15:0 | ND | ND |  |  |
|  | C16:0 | 0.03 ± 0.01 | 0.33 ± 0.27 |  | 0.388 |
|  | C18:0 | 0.02 ± 0.04 | 0.08 ± 0.06 |  | 0.411 |
|  | C20:0 | ND | ND |  |  |
|  | C21:0 | ND | ND |  |  |
|  | C22:0 | ND | ND |  |  |
|  | C23:0 | ND | ND |  |  |
|  | C24:0 | 0.01 ± 0.01 | 0.00 ± 0.00 |  | 0.284 |
|  | C14:1 ω5 | ND | ND |  |  |
|  | C15:1 ω5 | ND | ND |  |  |
|  | C16:1 ω7 | 0.01 ± 0.00 | 0.25 ± 0.25 |  | 0.422 |
|  | C16:1 ω9 | ND | ND |  |  |
|  | C17:1 ω7 | 0.00 ± 0.00 | 0.01 ± 0.01 |  | 0.443 |
|  | C18:1 ω7 | 0.01 ± 0.01 | 0.03 ± 0.03 |  | 0.397 |
|  | C18:1 ω9 | 0.03 ± 0.09 | 0.54 ± 0.45 |  | 0.372 |
|  | C20:1 ω9 | ND | ND |  |  |
|  | C22:1 ω9 | ND | ND |  |  |
|  | C24:1 ω9 | ND | ND |  |  |
|  | C18:2 ω6 | 0.01 ± 0.01 | 0.08 ± 0.07 |  | 0.432 |
|  | C20:2 ω6 | ND | ND |  |  |
|  | C22:2 ω6 | ND | ND |  |  |
|  | C18:3 ω3 | 0.00 ± 0.00 | 0.11 ± 0.11 |  | 0.428 |
|  | C18:3 ω6 | ND | ND |  |  |
|  | C20:3 ω3 | ND | ND |  |  |
|  | C20:3 ω6 | ND | ND |  |  |
|  | C20:4 ω6 | 0.01 ± 0.01 | 0.02 ± 0.02 |  | 0.401 |
|  | C22:4 ω6 | ND | ND |  |  |
|  | C20:5 ω3 | 0.01 ± 0.01 | 0.01 ± 0.01 |  | 0.458 |
|  | C22:5 ω3 | 0.01 ± 0.01 | 0.01 ± 0.01 |  | 0.443 |
|  | C22:5 ω6 | ND | ND |  |  |
|  | C22:6 ω3 | 0.01 ± 0.01 | 0.01 ± 0.01 |  | 0.454 |
| Plasma |  |  |  |  |  |
|  | C10:0 | ND | ND |  |  |
|  | C12:0 | ND | ND |  |  |
|  | C14:0 | 0.06 ± 0.02 | 0.20 ± 0.24 |  | **<0.01** |
|  | C15:0 | ND | ND |  |  |
|  | C16:0 | 2.26 ± 0.50 | 6.57 ± 0.58 |  | **<0.01** |
|  | C18:0 | 2.67 ± 0.29 | 4.82 ± 0.33 |  | **<0.01** |
|  | C20:0 | ND | ND |  |  |
|  | C21:0 | ND | ND |  |  |
|  | C22:0 | ND | ND |  |  |
|  | C23:0 | ND | ND |  |  |
|  | C24:0 | ND | ND |  |  |
|  | C14:1 ω5 | ND | ND |  |  |
|  | C15:1 ω5 | ND | ND |  |  |
|  | C16:1 ω7 | 0.26 ± 0.06 | 0.55 ± 0.04 |  | **0.01** |
|  | C16:1 ω9 | ND | ND |  |  |
|  | C17:1 ω7 | ND | ND |  |  |
|  | C18:1 ω7 | 0.33 ± 0.07 | 0.85 ± 0.08 |  | **<0.01** |
|  | C18:1 ω9 | 4.30 ± 0.45 | 6.77 ± 0.53 |  | **0.015** |
|  | C20:1 ω9 | ND | ND |  |  |
|  | C22:1 ω9 | ND | ND |  |  |
|  | C24:1 ω9 | ND | ND |  |  |
|  | C18:2 ω6 | 2.79 ± 0.43 | 4.48 ± 0.34 |  | **<0.01** |
|  | C20:2 ω6 | 0.11 ± 0.02 | 01.9 ± 0.03 |  | 0.068 |
|  | C22:2 ω6 | ND | ND |  |  |
|  | C18:3 ω3 | 0.44 ± 0.08 | 0.16 ± 0.04 |  | 0.017 |
|  | C18:3 ω6 | 0.01 ± 0.01 | 0.01 ± 0.01 |  | 0.632 |
|  | C20:3 ω3 | ND | ND |  |  |
|  | C20:3 ω6 | 0.07 ± 0.02 | 0.14 ± 0.02 |  | 0.021 |
|  | C20:4 ω6 | 1.28 ± 0.16 | 1.74 ± 0.14 |  | 0.057 |
|  | C22:4 ω6 | 0.07 ± 0.01 | 0.13 ± 0.02 |  | 0.02 |
|  | C20:5 ω3 | 0.25 ± 0.04 | 0.07 ± 0.03 |  | 0.019 |
|  | C22:5 ω3 | 0.14 ± 0.02 | 0.30 ± 0.04 |  | 0.018 |
|  | C22:5 ω6 | 0.01 ± 0.01 | 0.03 ± 0.05 |  | **<0.01** |
|  | C22:6 ω3 | 0.06 ± 0.02 | 0.31 ± 0.12 |  | 0.068 |

Table S2: Arithmetic means (‘Means’) and standard errors (‘SE’) of proportions of specific fatty acids (‘FA’) among saturated FA, monounsaturated FA or polyunsaturated FA in white adipose tissue (‘WAT’), muscle tissue (‘Muscle’) and blood plasma (‘Plasma’) of bears during the summer active period (‘Summer’) and in winter hibernation (‘Winter’). Differences of least square means (‘Lsmeans’) between seasons and p-values result from linear-mixed effects models (LMM). Sample sizes used in the LMM are presented in Table 1. Significant p-values are highlighted in bold. ‘ND’ refers to non-detectable.

| Tissues | Fatty acids | Means ± SE | | Winter-summer differences (%FA) | |
| --- | --- | --- | --- | --- | --- |
|  |  | **Summer** | **Winter** | **Lsmeans**± **SE** | **P-values** |
| WAT |  |  |  |  |  |
|  | C10:0 | ND | ND |  |  |
|  | C12:0 | ND | ND |  |  |
|  | C14:0 | 1.64 ± 0.80 | 2.80 ± 0.90 | 1.16 ± 0.99 | 0.300 |
|  | C15:0 | ND | ND |  |  |
|  | C16:0 | 59.95 ± 3.55 | 70.82 ± 3.95 | 10.87 ± 4.30 | 0.057 |
|  | C18:0 | 37.67 ± 3.43 | 25.78 ± 3.88 | -11.88 ± 5.75 | 0.063 |
|  | C20:0 | 0.74 ± 0.30 | 0.77 ± 0.33 | 0.03 ± 0.31 | 0.935 |
|  | C21:0 | ND | ND |  |  |
|  | C22:0 | 0.00 ± 0.06 | 0.10 ± 0.07 | 0.10 ± 0.09 | 0.321 |
|  | C23:0 | ND | ND |  |  |
|  | C24:0 | ND | ND |  |  |
|  | C14:1 ω5 | ND | ND |  |  |
|  | C15:1 ω5 | ND | ND |  |  |
|  | C16:1 ω7 | 0.62 ± 0.32 | 0.57 ± 0.34 | -0.05 ± 0.30 | 0.865 |
|  | C16:1 ω9 | 6.05 ± 0.92 | 9.65 ± 1.01 | 3.60 ± 1.05 | 0.020 |
|  | C17:1 ω7 | ND | ND |  |  |
|  | C18:1 ω7 | 5.62 ± 0.41 | 4.29 ± 0.47 | -1.33 ± 0.59 | 0.080 |
|  | C18:1 ω9 | 87.04 ± 1.05 | 84.12 ± 1.15 | -2.92 ± 1.10 | 0.050 |
|  | C20:1 ω9 | 0.67 ± 0.28 | 1.21 ± 0.32 | 0.54 ± 0.41 | 0.250 |
|  | C22:1 ω9 | ND | ND |  |  |
|  | C24:1 ω9 | ND | ND |  |  |
|  | C18:2 ω6 | 50.23 ± 3.43 | 58.03 ± 3.86 | 7.80 ± 4.64 | 0.154 |
|  | C20:2 ω6 | 0.00 ± 1.15 | 4.54 ± 1.35 | 4.54 ± 1.77 | 0.061 |
|  | C22:2 ω6 | ND | ND |  |  |
|  | C18:3 ω3 | 10.49 ± 2.54 | 18.78 ± 2.72 | 8.29 ± 2.08 | 0.023 |
|  | C18:3 ω6 | ND | ND |  |  |
|  | C20:3 ω3 | 0.00 ± 0.00 | 0.20 ± 0.13 | 0.20 ± 0.18 | 0.321 |
|  | C20:3 ω6 | 0.30 ± 0.26 | 1.45 ± 0.30 | 1.15 ± 0.37 | 0.028 |
|  | C20:4 ω6 | 30.56 ± 4.48 | 3.23 ± 5.26 | -27.24 ± 6.45 | **0.015** |
|  | C22:4 ω6 | 1.28 ± 0.85 | 2.39 ± 0.95 | 1.11 ± 1.03 | 0.333 |
|  | C20:5 ω3 | 1.89 ± 0.64 | 2.05 ± 0.66 | 0.17 ± 0.39 | 0.693 |
|  | C22:5 ω3 | 4.33 ± 1.24 | 5.11 ± 1.36 | 0.77 ± 1.84 | 0.684 |
|  | C22:5 ω6 | ND | ND |  |  |
|  | C22:6 ω3 | 3.00 ± 1.07 | 2.20 ± 1.13 | -0.80 ± 0.89 | 0.411 |
| Muscle |  |  |  |  |  |
|  | C10:0 | ND | ND |  |  |
|  | C12:0 | ND | ND |  |  |
|  | C14:0 | ND | ND |  |  |
|  | C15:0 | ND | ND |  |  |
|  | C16:0 | 62.44 ± 2.02 | 71.83 ± 1.78 | 9.39 ± 2.69 | **<0.01** |
|  | C18:0 | 37.55 ± 2.07 | 26.62 ± 1.66 | -10.94 ± 2.54 | 0.041 |
|  | C20:0 | ND | ND |  |  |
|  | C21:0 | ND | ND |  |  |
|  | C22:0 | ND | ND |  |  |
|  | C23:0 | ND | ND |  |  |
|  | C24:0 | 1.84 ± 1.29 | 0.00 ± 1.11 | -1.85 ± 1.60 | 0.273 |
|  | C14:1 ω5 | ND | ND |  |  |
|  | C15:1 ω5 | ND | ND |  |  |
|  | C16:1 ω7 | 2.92 ± 2.87 | 7.55 ± 2.53 | 4.63 ± 3.82 | 0.246 |
|  | C16:1 ω9 | ND | ND |  |  |
|  | C17:1 ω7 | 0.00 ± 0.00 | 0.59 ± 0.59 | 0.59 ± 0.59 | 0.443 |
|  | C18:1 ω7 | 14.15 ± 1.28 | 6.44 ± 1.09 | -7.71 ± 1.79 | **<0.01** |
|  | C18:1 ω9 | 83.25 ± 2.78 | 84.90 ±2.41 | 1.66 ± 3.29 | 0.625 |
|  | C20:1 ω9 | ND | ND |  |  |
|  | C22:1 ω9 | ND | ND |  |  |
|  | C24:1 ω9 | ND | ND |  |  |
|  | C18:2 ω6 | 73.81 ± 7.18 | 71.77 ± 6.19 | -2.04 ± 8.43 | 0.813 |
|  | C20:2 ω6 | ND | ND |  |  |
|  | C22:2 ω6 | ND | ND |  |  |
|  | C18:3 ω3 | -0.11 ± 5.63 | 6.02 ± 4.80 | 6.14 ± 6. 49 | 0.396 |
|  | C18:3 ω6 | ND | ND |  |  |
|  | C20:3 ω3 | ND | ND |  |  |
|  | C20:3 ω6 | ND | ND |  |  |
|  | C20:4 ω6 | 12.22 ± 4.01 | 17.72 ± 3.54 | 5.50 ± 5.35 | 0.322 |
|  | C22:4 ω6 | ND | ND |  |  |
|  | C20:5 ω3 | 0.32 ± 0.23 | 0.06 ± 0.20 | -0.26 ± 0.29 | 0.390 |
|  | C22:5 ω3 | 4.11 ± 1.64 | 2.24 ± 1.46 | -1.87 ± 1.63 | 0.296 |
|  | C22:5 ω6 | ND | ND |  |  |
|  | C22:6 ω3 | 10. 07 ± 2.16 | 2.85 ± 1.83 | -7.86 ± 2.70 | 0.020 |
| Plasma |  |  |  |  |  |
|  | C10:0 | ND | ND |  |  |
|  | C12:0 | ND | ND |  |  |
|  | C14:0 | 1.04 ± 0.18 | 1.69 ± 0.18 | 0.66 ± 0.25 | 0.017 |
|  | C15:0 | ND | ND |  |  |
|  | C16:0 | 42.57 ± 2.05 | 55.74 ± 2.05 | 13.18 ± 3.60 | **<0.01** |
|  | C18:0 | 56.46 ± 2.17 | 42.53 ± 2.17 | -13.93 ± 3.80 | **<0.01** |
|  | C20:0 | ND | ND |  |  |
|  | C21:0 | ND | ND |  |  |
|  | C22:0 | ND | ND |  |  |
|  | C23:0 | ND | ND |  |  |
|  | C24:0 | ND | ND |  |  |
|  | C14:1 ω5 | ND | ND |  |  |
|  | C15:1 ω5 | ND | ND |  |  |
|  | C16:1 ω7 | 4.96 ± 0.55 | 6.83 ± 0.55 | 1.88 ± 0.66 | 0.016 |
|  | C16:1 ω9 | ND | ND |  |  |
|  | C17:1 ω7 | ND | ND |  |  |
|  | C18:1 ω7 | 6.27 ± 0.80 | 10.55 ± 0.80 | 4.28 ± 1.20 | **<0.01** |
|  | C18:1 ω9 | 88.77 ± 1.27 | 82.66 ± 1.27 | -6.11 ± 1.75 | **<0.01** |
|  | C20:1 ω9 | ND | ND |  |  |
|  | C22:1 ω9 | ND | ND |  |  |
|  | C24:1 ω9 | ND | ND |  |  |
|  | C18:2 ω6 | 51.96 ± 2.22 | 59.08 ± 2.22 | 7.13 ± 3.38 | 0.064 |
|  | C20:2 ω6 | 2.20 ± 0.35 | 2.59 ± 0.35 | 0.39 ± 0.50 | 0.441 |
|  | C22:2 ω6 | ND | ND |  |  |
|  | C18:3 ω3 | 8.85 ± 1.21 | 2.26 ± 1.21 | -6.58 ± 1.99 | **<0.01** |
|  | C18:3 ω6 | 0.15 ± 0.07 | 0.18 ± 0.07 | 0.03 ± 0.10 | 0.766 |
|  | C20:3 ω3 | ND | ND |  |  |
|  | C20:3 ω6 | ND | ND |  |  |
|  | C20:4 ω6 | 27.34 ± 2.28 | 23.68 ± 2.28 | -3.65 ± 3.23 | 0.272 |
|  | C22:4 ω6 | 1.51 ± 0.22 | 1.75 ± 0.22 | 0.24 ± 0.30 | 0.435 |
|  | C20:5 ω3 | 4.92 ± 0.67 | 1.05 ± 0.67 | -3.87 ± 0.94 | **0.01** |
|  | C22:5 ω3 | 2.58 ± 0.33 | 3.94 ± 0.33 | 1.35 ± 0.47 | **0.01** |
|  | C22:5 ω6 | 0.10 ± 0.06 | 0.40 ± 0.06 | 0.30 ± 0.08 | **<0.01** |
|  | C22:6 ω3 | 1.24 ± 0.81 | 3.66 ± 0.81 | 2.42 ± 1.08 | 0.050 |
